# Pedigree reconstruction from SNP data: parentage assignment, sibship clustering and beyond

**DOI:** 10.1111/1755-0998.12665

**Published:** 2017-04-06

**Authors:** Jisca Huisman

**Affiliations:** ^1^ Ashworth Laboratories School of Biological Sciences Institute for Evolutionary Biology University of Edinburgh Edinburgh EH9 3FL UK

**Keywords:** parentage assignment, pedigree, sequoia, sibship clustering, single nucleotide polymorphism

## Abstract

Data on hundreds or thousands of single nucleotide polymorphisms (SNPs) provide detailed information about the relationships between individuals, but currently few tools can turn this information into a multigenerational pedigree. I present the r package sequoia, which assigns parents, clusters half‐siblings sharing an unsampled parent and assigns grandparents to half‐sibships. Assignments are made after consideration of the likelihoods of all possible first‐, second‐ and third‐degree relationships between the focal individuals, as well as the traditional alternative of being unrelated. This careful exploration of the local likelihood surface is implemented in a fast, heuristic hill‐climbing algorithm. Distinction between the various categories of second‐degree relatives is possible when likelihoods are calculated conditional on at least one parent of each focal individual. Performance was tested on simulated data sets with realistic genotyping error rate and missingness, based on three different large pedigrees (*N *=* *1000–2000). This included a complex pedigree with overlapping generations, occasional close inbreeding and some unknown birth years. Parentage assignment was highly accurate down to about 100 independent SNPs (error rate <0.1%) and fast (<1 min) as most pairs can be excluded from being parent–offspring based on opposite homozygosity. For full pedigree reconstruction, 40% of parents were assumed nongenotyped. Reconstruction resulted in low error rates (<0.3%), high assignment rates (>99%) in limited computation time (typically <1 h) when at least 200 independent SNPs were used. In three empirical data sets, relatedness estimated from the inferred pedigree was strongly correlated to genomic relatedness.

## Introduction

Pedigrees have many uses in a wide variety of fields, ranging from animal breeding and human genealogy to wildlife genetics and ethology. Parentage assignment remains essential for unbiased estimation of trait heritabilities, as even though pairwise relatedness coefficients can now be estimated more precisely directly from genomic data than from a pedigree (Visscher *et al*. 2006; Bérénos *et al*. 2014), heritability estimates still require proper accounting for the similarity due to shared parents (Kruuk & Hadfield 2007; Bérénos *et al*. 2014). The relevant shared parent is unobservable in many marine species, den‐sharing social mammals or seed‐dispersing plants, and in such cases, a pedigree is required to distinguish parents from full‐siblings and offspring, or between paternal and maternal half‐siblings. Moreover, in natural populations, pedigrees provide estimates of reproductive success, the key indicator of individual fitness. Thus, pedigree reconstruction remains useful in the current genomics era.

A plethora of methods have been developed to reconstruct pedigrees based on a dozen or so multi‐allelic microsatellites (see Jones *et al*. (2010) for an overview). High‐resolution SNP data can open up new ways of pedigree reconstruction, by utilizing the more reliable distinction between different categories of relatives. Simultaneously, the lower information content per SNP necessitates a large number of markers to obtain the same accuracy as with a dozen microsatellites. This puts a considerable strain on machinery intended to deal with variable number of alleles per marker, while the binary nature of typical SNPs allows some computational short cuts to be taken. For example, dealing with genotyping errors and missing data requires summation of probabilities over all possible actual genotypes (Wang 2004; Hadfield *et al*. 2006. For an offspring–mother–father trio, there are 3^3^ = 27 possible genotype combinations per SNP, and all probabilities for each locus are easily calculated once and stored in look‐up tables. This is less practical for a microsatellite locus with say 10 alleles and (10^2^ + 10)^3^/2 = 166 375 possible trio genotypes, and alternative tactics have been developed (e.g. Wang 2004). Therefore, new tools are required, specifically designed for SNPs.

Pedigree reconstruction not only entails parentage assignment, but when sampling of candidate parents is incomplete, also clustering of (half‐)siblings sharing the same, nongenotyped parent. This is often performed using colony (Wang 2004, 2012; Jones & Wang 2010), and can substantially increase the number of within‐cohort pedigree links (e.g. Walling *et al*. 2010). However, amalgamating sibships across multiple cohort is not straightforward, and reconstructed sibships are typically unconnected to earlier parts of the pedigree, affecting amongst others estimates of inbreeding coefficients (Taylor *et al*. 2015). Assigning grandparents to sibship clusters would overcome the latter limitation and involves highly similar comparisons to assigning half‐siblings. To my knowledge, this is not attempted in any available software, although methods to assign grandparents to individuals have been described (e.g. Letcher & King 2001; VanRaden *et al*. 2013).

### Pedigree reconstruction methods

Most pedigree reconstruction methods can be grouped into three broad categories: exclusion methods, relatedness‐based methods and likelihood‐based methods, which are of increasing power, but have increasing computational cost as a trade‐off. The first simply excludes all candidate parents which do not share at least one allele with the focal individual at each marker locus, and has been used with both microsatellites (see Thompson & Meagher 1987) and SNP data (Calus *et al*. 2011; Hayes 2011). Often some genotyping errors or mutations are allowed for, and the main advantage is that it is very fast. When a very large number of SNPs are used, the number of opposing homozygotes can also be used to differentiate full‐siblings and half‐siblings from unrelated pairs (Calus *et al*. 2011). The major caveat is that when several candidate parents are nonexcluded, other methods are required to differentiate between them.

Methods in the second category estimate pairwise relatedness *r* or kinship coefficients between individuals, and use these to categorize the data into first‐degree relatives, second‐degree relatives and unrelated (Thompson 1975). In systems with nonoverlapping generations and no inbreeding, this may be sufficient to fully reconstruct a pedigree. When generations overlap, different statistics are required to differentiate between parent–offspring pairs and full‐siblings, for example, which are both related by *r* = 0.5. Parent–offspring and full‐sibling pairs can be distinguished using the Cotterman coefficients, the probabilities that the pair share 0, 1 or 2 alleles identical by descent at a locus, but neither pairwise measure can distinguish between half‐siblings, grandparents and full aunts/uncles (all *r* = 0.25).

In comparison, likelihood methods (the third category) are considerably more powerful (Thompson 1986; Hill *et al*. 2008), although computationally notably slower. The likelihood of a particular pedigree configuration is the probability of observing the observed genotypes, conditional on the genotypes of the assigned parents, multiplied over all individuals and, when loci are assumed independent, multiplied over all loci. This approach makes use of heterozygous genotypes, which are ignored by exclusion methods, and can be calculated over many individuals jointly, whereas relatedness is typically calculated pairwise (although see Wang (2007) for a triadic version). Likelihoods allow more powerful distinction between alternative candidate fathers when one can condition on the genotype of a known mother, as implemented in cervus (Marshall *et al*. 1998), colony (Wang 2004) and masterbayes (Hadfield *et al*. 2006), amongst others. Likelihood calculations that condition on at least one parent each of a pair of individuals can distinguish between the three types of second‐degree relatives (see Methods in Appendix S1, Supporting information), which is impossible when considering only the genotypes of the two focal individuals and (presumed) unlinked markers (Epstein *et al*. 2000).

### Likelihood maximization

Maximizing the total likelihood over all individuals is challenging, as the number of possible pedigree configurations increases quickly with the number of individuals. A common way to reduce computational cost is to consider only pairwise likelihoods, and find the most likely parent(s) for each individual in turn (e.g. cervus, Marshall *et al*. 1998). One caveat with this is that close relatives who are not parent and offspring (not PO) may have a higher pairwise likelihood to be PO than to be unrelated (U) and thus a positive log‐likelihood ratio Λ_PO/U_ (Thompson 1986). To put it differently, when PO and U are not the only possible alternatives, rejecting hypothesis U is not equivalent to accepting PO, and Λ_PO/U_ is no longer the most powerful test statistic (the Neyman–Pearson lemma, Anderson & Garza 2006). Consequently, there is often considerable overlap in the distribution of Λ_PO/U_ of true PO pairs and other types of relatives (Thompson & Meagher 1987; Marshall *et al*. 1998). Those true full‐siblings who have at least one allele in common at every locus have a higher expected Λ_PO/U_ than parent–offspring pairs, but have an even higher expected likelihood to be full‐siblings (Thompson & Meagher 1987). Therefore, while Λ_PO/U_ and Λ_PO/FS_ are necessarily highly correlated, each provides information that the other does not (Thompson 1986).

Thus, one solution to ensure that one indeed maximizes the total likelihood is to calculate for each set of candidate relatives the likelihoods under many possible alternative relationships. This is implicit to kinship (Goodnight & Queller 1999) and has been implemented for parentage assignment in franz (Riester *et al*. 2009), and is implemented more comprehensively here. One reason for the limited implementation of this approach with microsatellites is the large computational costs involved with calculating likelihoods of many relationship alternatives over the very large number of possible true genotypes. Moreover, with a typical number of 10–20 microsatellites, it is nearly infeasible to distinguish reliably between the various relationship classes. In contrast, with a large number of SNPs, different relationships can be distinguished reliably.

Inbred and complex bilineal relationships (see Fig. 10) are often excluded from consideration, to keep computations feasible and tractable (Goodnight & Queller 1999; Wang 2004; Jones & Wang 2010; Anderson & Ng 2016) However, pedigree reconstruction in small populations is regularly performed with the specific aim to study the amount of inbreeding. Moreover, in a range of mammal species, female relatives live together and are therefore likely to mate with the same male (Stopher *et al*. 2012, and references therein). The resulting offspring are related by more than *r* = 0.25 and can therefore easily be misclassified as full‐siblings when full‐sibling, half‐sibling and unrelated are the only alternatives considered.

**Figure 1:**
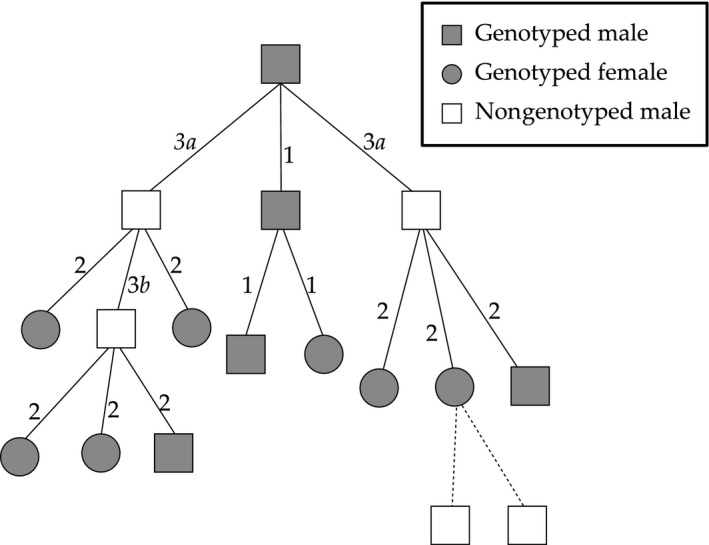
Example part pedigree with only paternal links shown. Abbreviations indicate when the link is inferred: during (*1*) parentage assignment, (*2*) sibship clustering (assignment of a dummy parent), (*3a*) assignment of genotyped grandparents to sibships, (*3b*) assignment of dummy individuals as grandparents to other sibships, or (dashed) based on nongenetic data only (not by sequoia). Note that links *3a* and *3b* are not inferred by other programs, which would result in four unconnected pedigree fragments.

Here, I present an algorithm that compares likelihoods for seven different relationship alternatives, including their inbred derivatives, speeded up by steps to exclude unlikely relatives. It (1) assigns parents, (2) clusters sibling groups across multiple cohorts, (*3a*) assigns grandparents to sibships and singletons and (*3b*) identifies avuncular links between sibships (Fig. 1), using presumed independent SNPs. Pedigree inference based on the length and distribution of genome segments shared between individuals is theoretically a more powerful approach (Hill & White 2013), but for many species, a reliable linkage map is not (yet) available. Performance of sequoia is illustrated on simulated data sets from three different pedigree structures, and empirical data sets from wild red deer (*Cervus elaphus*), great tits (*Parus major*) and domestic pigs (*Sus scrofa*). I show that several hundred independent SNPs with high minor allele frequency are sufficient to obtain a high assignment rate (>99%) and low error rate (<0.1%).

## Methods

### Overview

**Figure 2:**
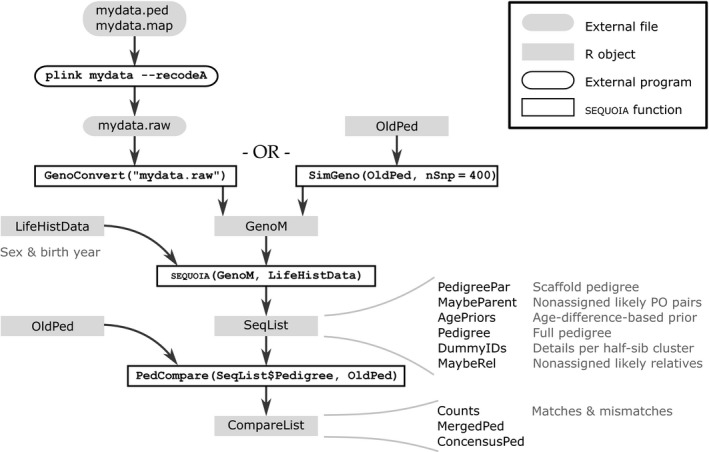
Overview of program use. Input consists of a numeric matrix with genotypes either converted from standard plink format or simulated from a pedigree, and a dataframe with life‐history data (ID, sex and birth year), and output of an r list with the pedigree and various other elements. A detailed manual is given in the r vignette.

The input format for sequoia is easily obtained from a genotype file in standard plink format (Fig. 2, details in r vignette) and should be provided together with sex and birth year information for the majority of genotyped individuals.

When sequoia is called, first a check for duplicate identities and genotypes is performed to avoid downstream problems. Next, several iterations of parentage assignment are performed, until the total likelihood (defined in Eqn 1 below) asymptotes. This provides a robust, conservative ‘pedigree scaffold’, as distinguishing parents from nonparents has a lower false‐positive rate than distinguishing between various other classes of relatives (see Results). The pedigree scaffold is returned for user inspection, to check for swapped or mislabelled samples, for example. In addition, a list is returned of identified parent–offspring pairs for which polarity could not determined, due to absent or incompatible age or sex information.

Then, clusters of half‐siblings with an unsampled parent are found and assigned a ‘dummy’ parent. Subsequently, parents may get assigned to these dummy individuals, providing pedigree links across generations. This is again done in an iterative fashion. Alternative orders of the various steps were explored but resulted in higher error rates (see Appendix S1, Supporting information).

Biological feasibility of the resulting pedigree is achieved by ensuring that, given the current pedigree, (i) an individual cannot be its own ancestor; (ii) ancestors are born prior to their descendants, or either or both have an unknown birth year; and (iii) the two parents of an individual are of opposite sex, or either one is of unknown sex (i.e. no hermaphrodites or asexual reproduction allowed and thus no selfing).

### Filtering steps

Use of opposite homozygosity as a filtering step is a computationally fast method to dramatically reduce the number of potential parent–offspring (PO) pairs (Hill *et al*. 2008; Hayes 2011; Anderson 2012). By default, a liberal threshold of *T*_OH_ = 3 + *εL* is used to avoid exclusion of true PO pairs, where *L* is the number of loci and *ε* the per‐locus genotyping error rate. Typically some pairs of non‐PO close relatives will be nonexcluded, particularly full‐sibling (FS) pairs (see Calus *et al*. 2011).

A second filtering step for parentage assignment, and the only filtering step for the other stages, consists of calculating the log‐likelihood ratio $\Lambda_{R/U}^{*}$ between the focal relationship *R* and unrelated U, without conditioning on the parents in the current pedigree to simplify and speed up computations. A liberal, log‐scale negative threshold (the user‐adjustable *T*_Filter_) is used to again avoid exclusion of true relatives.

### Parentage assignment

For each individual in turn, from earliest born to last born to unknown birth years, all individuals with which the focal individual is nonexcluded as a PO pair and which are older or of unknown age difference are considered as candidate parents, and the likelihoods for the seven alternative relationships are calculated (Table 1, $L_{H0}$–$L_{H6}$). If the focal relationship *R* (here PO) has a higher likelihood than the most likely alternative relationship (denoted by ∨  for brevity), by a user‐defined margin *T*_assign_, an assignment is made (Λ_R/∨_ > *T*_assign_; glossary provided in Table 2). If there are multiple candidate parents, these likelihoods are calculated for all possible opposite‐sex candidate parent pairs and all possible single candidate parents (details in Appendix S1, Supporting information). Parent assignments are made according to the highest likelihood, which may include removal of earlier‐assigned parents. This approach maximizes assignment rate and minimizes the chance that, for example, full‐siblings or double‐grandparents are assigned as parents.

**Table 1:** Genealogical relationships considered in this article, and their mean pairwise relatedness *r* in absence of inbreeding or additional relationships between the pair of individuals

	Relationship	Code	Mean *r*
*H*_1_	Parent‐offspring	PO	1/2
*H*_2_	Full‐siblings	FS	1/2
*H*_3_	Half‐siblings	HS	1/4
	Maternal siblings (full or half)	MS	1/2 or 1/4
	Paternal siblings (full or half)	PS	1/2 or 1/4
*H*_4_	Grandparent–grand‐offspring	GG	1/4
*H*_5_	Full aunt/uncle–niece/nephew	FA	1/4
*H*_6*a*_	Half aunt/uncle–niece/nephew	HA	1/8
*H*_6*b*_	Great‐grandparent–great‐grand‐offspring	GGG	1/8
*H*_6*c*_	Full cousins	CC	1/8
*H*_0_	Unrelated	U	0

Double full first cousins (*r* = 1/4) are currently not explicitly considered

**Table 2:** Glossary

	Definition
*A*	Focal individual
***A***	Focal sibship (group of half‐siblings)
*D*_*A*_	Mother (Dam) of focal individual
*k*	Parent or sibship type; maternal or paternal
*l*	Locus
*P*_*ε*_	Genotyping error term
*P*_*M*_	Mendelian inheritance term
*P*_*P*_	Parental probability term
*R*	Focal relationship
*S*_*A*_	Father (Sire) of focal individual
*T*_assign_	Threshold Λ_R/∨_ for assignments
*T*_filter_	Threshold for $\Lambda_{R/U}^{*}$ to differentiate ‘possibly relatives’ from ‘certainly not relatives’
*X*	Observed genotype
*x*	Actual genotype
∨	Most likely alternative relationship
$L_{H0}$	Likelihood under *H*_0_
$\Lambda_{R/U}^{*}$	Likelihood ratio, does not condition on current parents
Λ_*R*/∨_	Likelihood ratio, does condition on current parents

### Likelihood calculations

The quantity that is maximized is the total likelihood L of the pedigree configuration ***P*** over all *N* genotyped individuals,

(eqn 1) $$L\left(P\right)=\prod_{A=1}^{N}L\left(A,D_{A},S_{A}\right)\approx \prod_{A=1}^{N}\prod_{l}P\left(A_{l}=X|D_{A},S_{A}\right),$$

where *P*(*A*_*l*_ = *X*|*D*_*A*_, *S*_*A*_) is the probability of observing genotype *X* at locus *l* in individual *A*, conditional only on its parents *D*_*A*_ and *S*_*A*_ in pedigree ***P***. It is assumed a set of SNPs is used which are unlinked and in low linkage disequilibrium, such that a simple multiplication over all loci provides a good approximation of the total likelihood.

The probability *P*(*A*_*l*_ = *X*|*D*_*A*_, *S*_*A*_) can be broken down into a genotyping error term *P*_*ε*_, a Mendelian inheritance term *P*_*M*_ (denoted transmission probability *T* in Meagher (1986) and Marshall *et al*. (1998)) and a parental genotype probability term *P*_*P*_:

(eqn 2) $$\begin{array}{cc} P\left(A_{l}=X|D_{A},S_{A}\right)=\sum_{x}\sum_{y}\sum_{z}P_{\epsilon }\left(A_{l}=X|A_{l}=x,\epsilon \right) & \\ \times P_{M}\left(A_{l}=x|D_{A_{l}}=y,S_{A_{l}}=z\right)P_{P}\left(D_{A_{l}}=y\right)P_{P}\left(S_{A_{l}}=z\right). \end{array}$$

The first term (*P*_*ε*_) is a function of A's actual genotype *x* and the genotyping error rate *ε*, which is assumed constant across loci. Details of the genotyping error model are given in Methods in Appendix S1 (Supporting information). The second term (*P*_*M*_) is the probability that individual *A* inherited actual genotype *x* from its parents *D*_*A*_ and *S*_*A*_, conditional on their actual genotypes *y* and *z*. This probability can take values of 0, 1/4, 1/2 and 1. As SNP genotypes can only take three possible values (0, 1 or 2 copies of the minor allele), the likelihood components *P*_*ε*_ and *P*_*M*_ can be calculated once at initiation and stored in look‐up tables, for increased computational efficiency. In contrast, the parental genotype probabilities *P*_*P*_ (the third term) are continuously updated. They give the probability that *A*'s parents carry actual genotypes *y* and *z* and come in three different flavours, denoted by a superscripted prefix:

$$P_{P}=\left\{\begin{array}{c} {}^{h}P_{P}\text{for an unknown parent;}\\ {}^{g}P_{P}\text{for a known, genotyped parent;}\\ {}^{d}P_{P}\text{for a dummy parent.} \end{array}\right.$$

When say parent *D*_*A*_ is unknown, ^*h*^ *P*_*P*_(*D*_*A*_ = *y*|*q*_*l*_) takes the standard values when assuming Hardy–Weinberg equilibrium of $q_{l}^{2}$, 2*q*_*l*_(1 − *q*_*l*_) and (1 − *q*_*l*_)^2^, that is unknown parents are assumed a random draw from the population. When *D*_*A*_ is a known genotyped individual, the probabilities for all possible actual genotypes *y* are calculated conditional on *D*_*A*_'s observed genotype *Y* and its parents, if any. Using that *P*(*A*|*B*) = *P*(*B*|*A*)*P*(*A*)/*P*(*B*) (Bayes’ theorem) and dropping subscripts *l* for brevity,

(eqn 3) $${}^{g}P_{P}\left(D_{A}=y|D_{A}=Y,D_{D_{A}},S_{D_{A}}\right)=\frac{P_{\epsilon }\left(D_{A}=Y|D_{A}=y\right)P_{M}\left(D_{A}=y|D_{D_{A}},S_{D_{A}}\right)}{P(D_{A}=Y)},$$

where

$$P\left(D_{A}=Y\right)=\sum_{y^{\prime }}P_{\epsilon }\left(D_{A}=Y|D_{A}=y^{\prime }\right)P_{M}\left(D_{A}=y^{\prime }|D_{D_{A}},S_{D_{A}}\right)),$$

and $D_{D_{A}}$ and $S_{D_{A}}$ are grandparents of *A*. When *D*_*A*_ is not genotyped at a particular locus, the term *P*_*ε*_(*D*_*A*_ = *Y*|*D*_*A*_ = *y*) is omitted from Eqn 3, and ^*g*^ *P*_*P*_(*D*_*A*_ = *y*) becomes dependent on the grand‐parental genotypes only. When both $D_{D_{A}}$ and $S_{D_{A}}$ are unknown, ^*g*^ *P*_*P*_(*D*_*A*_ = *y*) reduces further to ^*h*^ *P*_*P*_(*D*_*A*_ = *y*|*q*_*l*_). The probability ^*d*^ *P*_*P*_ for dummy parents is defined in the section ‘Sibship likelihoods’ (Eqn 5).

**Figure 3:**
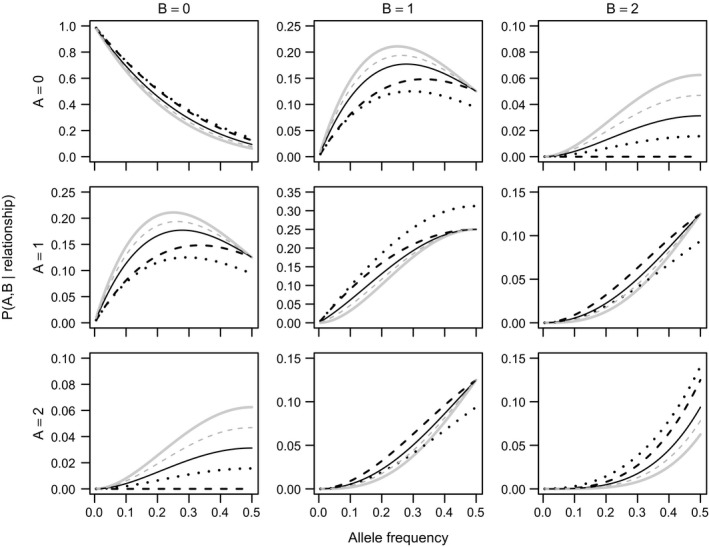
Single‐locus probability of observing genotypes *A* and *B* (0, 1 or 2 copies of the minor allele) as a function of the minor allele frequency *q*, under the hypotheses U (solid grey line), PO (dashed black), FS (dotted black), HS, GG or FA (solid black, indistinguishable from each other), or HA or GGG (dashed grey) (Equations S2–S11 in Appendix S1, Supporting information).

*P*_*ε*_, *P*_*M*_ and *P*_*P*_ can be combined to calculate the likelihood of observing the genotypes of a group of individuals (*n* ≥ 1) under any relationship configuration. Single‐locus likelihoods are illustrated in Fig. 3 for the special case of two focal individuals *A* and *B*, when neither individual has any parent yet assigned. In this case, second‐degree relatives (HS, GG and FA) cannot be distinguished from each other. However, when one can condition on the genotype of a parent or dummy parent of each individual, such a distinction can be made. Details on these likelihood equations, and those for inbred relationships, are given in Appendix S1 (Supporting information).

### Sibship clustering

A sibship is here defined as a group of half‐siblings sharing an unsampled parent, containing zero or more sets of full‐siblings. During each iteration of sibship clustering, first all pairs of likely HS and FS are identified using $\Lambda_{HS/U}^{*}>T_{filter}$, followed by calculation of $L_{H0}$–$L_{H6}$ for the pair. These pairs are clustered into sibships using likelihoods calculated over the pair and all putative siblings. Assignments are made when (max (Λ_HS/*∨*_, Λ_FS/*∨*_) >*T*_assign_. Subsequently during each iteration, all sibships of the same type are considered for merging to minimize erroneous splitting of true sibships, and all individuals who lack a parent of type *k* are considered for addition to each sibship of type *k* to maximize assignment rate (Methods in Appendix S1, Supporting information).

### Sibship likelihood equations

The marginal likelihood of sibship ***A*** in absence of inbreeding is

(eqn 4) $$\begin{array}{cc} L(A|D_{A}=x) & =\prod_{l}\sum_{v}\sum_{w}P_{M}\left(D_{A}=x|D_{D_{A}}=v,S_{D_{A}}=w\right)P_{P}\left(D_{D_{A}}=v\right)P_{P}\left(S_{D_{A}}=w\right)\\ & \times \prod_{i=1}^{n_{A}}\sum_{y_{i}}P_{P}\left(S_{i}=y_{i}\right)\prod_{j=1}^{m_{A,i}}\sum_{z}P_{\epsilon }\left(A_{i,j}=Z|A_{i,j}=z,\epsilon \right)P_{M}\left(A_{i,j}=z|D_{A}=x,S_{i}=y_{i}\right), \end{array}$$

where * S*_*i*_ is the parent of full‐siblings $A_{i,1}\ldots A_{i,m_{A,i}}$, *S*_*i*_ of opposite sex than *D*_***A***_, and sibship ***A*** consists of *n*_*A*_ full‐sib families. This is a standard expression, used by for example colony (Wang 2004; Eqn 3) and Fullsniplings (Anderson & Ng 2016, implicit). A more general expression allowing for inbreeding (Equation S16 in Appendix S1, Supporting information) is implemented in the algorithm.

The parental probability ^*d*^ *P*_*P*_ is then calculated as

(eqn 5) $${}^{d}P_{P}\left(D_{A}=x\right)=\frac{L(A|D_{A}=x)}{\sum_{x^{\prime }}L\left(A|D_{A}=x^{\prime }\right)}.$$

Note that when *S*_*i*_ also is a dummy parent, ^*d*^ *P*_*P*_(*S*_*i*_ = *y*_*i*_) in Eqn (4) is calculated without the contribution of the joined offspring *A*_*i*_, to avoid double counting. Most often, the joined likelihood over ***A*** and all directly connected sibships is calculated, as *P*_*P*_(*S*_*i*_ = *y*_*i*_) will be a function of the presumed genotype of *D*_***A***_, and therefore, the *P*_*P*_(*S*_*i*_ = *y*_*i*_)'s of different connected sibships are nonindependent (Methods in Appendix S1, Supporting information).

### Parents and grandparents of sibships

Initial parentage assignment may have been incomplete, for example when the true parent has an unknown birth year. Therefore, replacement of dummy parents by genotyped individuals is attempted for all sibships, as well as assignment of parents to singletons, as described above.

Lastly, in each iteration, grandparents are assigned, in a process similar to parentage assignment. This includes potential assignments of the dummy parent of one sibship (say *D*_***B***_) as the grandparent of sibship ***A***, when *D*_***B***_ is more likely to be the grandparent of $A_{1},A_{2},\ldots ,A_{n_{A}}$ than related in any of the alternative ways listed in Table 1). To minimize false positives, grandparent assignment to sibship is conducted from the second iteration onwards, and assignment to singletons from the third iteration onwards; this should not prevent assignment of any true grandparents (Results: Algorithm order in Appendix S1, Supporting information). Grand‐offspring–grandparent pairs are treated as sibship clusters with a single member, to which additional siblings may be added in subsequent iterations.

### Age information

The age difference between individuals can be highly informative to distinguish between, for example, parents and full‐siblings, or between grandparents and half‐siblings. sequoia makes use of an age‐difference‐based prior, which in its simplest form is an indicator whether a given relationship is possible (1) or not (0) given the age difference between the two individuals. After parentage assignment, the empirical age distribution of fathers and mothers and between maternal and paternal siblings is used as prior to assist subsequent sibship clustering (Methods in Appendix S1, Supporting information). For each hypothesized relationship, the genetic‐based likelihoods are multiplied by these age‐difference‐based prior probabilities, that is genotypes and age differences are treated as independent sources of information. Methods are implemented to deal with missing age or sex information (Methods in Appendix S1, Supporting information).

### Assignment confidence

In the returned pedigree, a value Λ_PO/*∨*_ is associated with each assigned parent and dummy parent, which is the log10 likelihood ratio between the candidate parent being the parent and the most likely alternative relationship, calculated conditional on all other pedigree links. The Λ_PO/∨_ for the parent pair is calculated relative to the highest likelihood scenario with one or neither parent assigned. For dummy individuals, a similar approach is followed with respect to the sibship grandparents; calculations are always conditional on all its offspring. Assignment confidence is currently not expressed as a probability, but various *post hoc* approaches could be considered if these are required (see Results and Discussion in Appendix S1, Supporting information).

### Data sets

The algorithm was tested on simulated data sets generated from three different pedigree structures, described below, to give a general indication of assignment and error rates. For each pedigree, after simulation of genotype data (Methods in Appendix S1, Supporting information), a varying proportion of parental genotypes was discarded to assess sibship clustering. For all simulated data sets, 0.5% of per‐locus genotypes were set to missing, and 0.1% were replaced by a random genotype, which is a low but realistic error rate (Methods, see also Fig. S7 in Appendix S1, Supporting information).

In addition, the algorithm was run on empirical SNP data sets from red deer (Huisman *et al*. 2016), great tits (Santure *et al*. 2015), and pigs (Cleveland *et al*. 2012). In each case, plink (Purcell *et al*. 2007) was used to select 400–600 SNPs for pedigree inference, with minor allele frequency above 0.4 and in low linkage disequilibrium with each other.

#### Pedigree I: Full‐sib families

Pedigree I consisted of 1157 genotyped individuals in a single generation, divided over 432 full‐sib families (Table 3) with 1–11 individuals each (mean: 2.68, 143 singletons). It is identical to the pedigree structure used in Anderson & Ng (2016) to compare performance of colony (Wang 2012) and fullsnplings (Anderson & Ng 2016), and is derived from an empirical salmon data set.

**Table 3:** Total number of individuals in various categories for each Pedigree

	Pedigree I	Pedigree II	Pedigree III
Total	2021	1000	1998
Mother known	1157	960	1642
Father known	1157	960	1202
Unique mothers	432	80	462
Unique fathers	432	120	193

Pedigree I consists of a single generation of full‐sib families, Pedigree II of 5 discrete generations of full‐ and half‐sib families, and Pedigree III is the empirical pedigree of the 17 most recent birth year cohorts of a wild Red deer population.

#### Pedigree II: Multigenerational half‐sib

**Figure 4:**
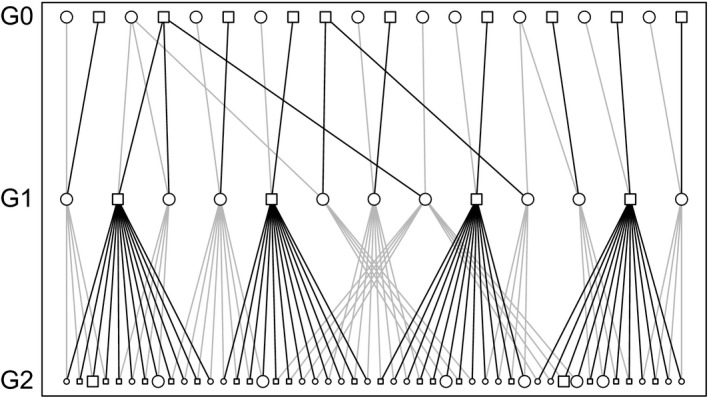
Mating scheme in Pedigree II, showing a subset of individuals selected to breed in G1, their parents (in G0) and their offspring (in G2), some of which are selected at random (larger symbols) to become parents of G3. Note that by chance, two full‐siblings are selected as mates (2nd and 3rd individual from the left in G1).

The second pedigree mimicked a small closed population and consisted of five nonoverlapping generations, with full‐sib families nested within interconnected half‐sib clusters. Each female mated with two random males and each male with three random females, producing four full‐sib offspring per mating (Fig. 4). Each generation, 24 female and 16 male breeders were drawn at random from the 192 offspring born. Matings between full or half‐siblings were allowed, and average inbreeding coefficient in the fifth generation was 0.053 (range: 0.008–0.289). This artificial pedigree is provided with the r package.

#### Pedigree III: Red deer

The third set of simulated data sets was based on the empirical pedigree of red deer detailed below. It consists of the last 17 birth year cohorts (1999–2015) and their parents, totalling 1998 individuals.

#### Empirical data set 1: Insular red deer

The pedigree from the study population of wild red deer on the Isle of Rum is characterized by extensively overlapping generations, matrilineal association of females, and numerous instances of close and moderate inbreeding (Clutton‐Brock *et al*. 1982). Each breeding season, immigration of males born elsewhere on the island occurs. The previous pedigree was reconstructed based on 9–15 microsatellite markers using masterbayes and colony (Walling *et al*. 2010), and includes 441 founders and 2340 nonfounders born up until 2012. The SNP data set used consisted of 2572 individuals born up until 2013 genotyped for 37 410 polymorphic autosomal SNPs (Huisman *et al*. 2016), of which 440 SNPs were used for pedigree inference.

#### Empirical data set 2: Pig breeding line

This data set was made available for comparing genomic prediction methods (Cleveland *et al*. 2012), and contained 3534 individuals with genotypes for 52 843 SNPs, of which 652 SNPs were used here. The provided pedigree consisted of 6473 individuals and included the parents and grandparents of genotyped individuals, where known. No birth year information is publicly available; therefore, the generation numbers in the provided pedigree (1 = founders, 2 = offspring of founders, 3 = offspring of g2 or g2 × founders, etc.) were treated as cohorts.

#### Empirical data set 3: Wild great tit

The second data set was the larger of the two data sets used for a study on the genetic architecture of quantitative traits by Santure *et al*. (2015), from a open population of great tits in Oxfordshire. It consisted of genotype data for 2497 individuals on 5592 SNPs, of which 488 SNPs were used here. The provided social pedigree included 1035 founders and 1674 nonfounders, and birth year data for 1558 individuals was extracted from the excel file with phenotypic data.

#### Comparison to other software

sequoia's performance was compared to that of colony 2.0.6.1 (Wang 2013), using its full‐likelihood–pair‐likelihood score combined (FPLS) analysis method, with otherwise default settings: without inbreeding (as recommended by the colony user guide when the inbreeding level is not high), medium run length, weak sibship size priors of 1.0, and with sibship scaling.

In addition, the program franz (Riester *et al*. 2009) was run, which performs parentage assignment only, optionally assisted by clustering of full‐siblings. Lenient settings were used throughout, with a maximum number of candidate parents of 500, reproductive ages of females and males between 1 and 20, and otherwise default settings.

Lastly, exclusion based on the number of opposing homozygous loci was evaluated as a parentage assignment method, assigning the first nonexcluded parent of each sex. The same allowance for genotyping errors was used as in sequoia, of maximum 3 mismatching loci.

#### Assignment and error rates

The assignment rate (AR) for the simulated data sets was calculated as the number of individuals with a correctly inferred parent, divided by *N*_*k*_, the number of individuals with a parent of sex *k* in the true pedigree, averaged over maternal and paternal links. The error rate (ER) was calculated as the fraction of the total number of individuals (founders + nonfounders) with an incorrectly assigned parent. A sibship parent, say dummy father, was deemed correct if the majority (>50%) of inferred paternal siblings (PS) were true PS. For both erroneous merging and erroneous nonmerging, the error count equalled the size of the smaller of the two sibships.

## Results

### Distribution of Λ

**Figure 5:**
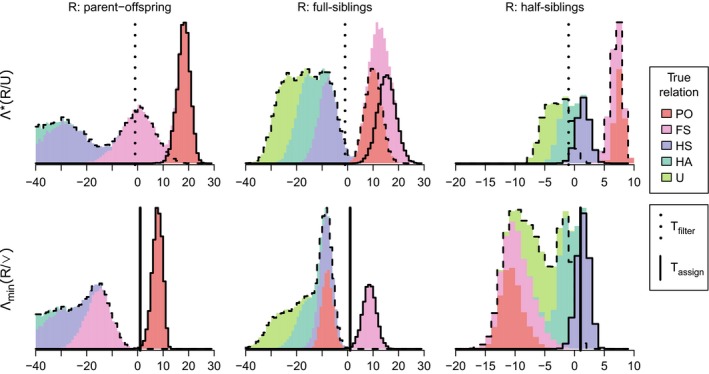
Pairs truly related according to a focal relationship (headers, solid outline) are more clearly distinguished from other related pairs (dashed outline) using Λ_R/∨_ (bottom row) than when using Λ_R/U_ (top). Likelihoods are not conditional on any parental genotypes for PO (left) and FS (middle), and conditional on the genotypes of one parent each for HS (right) (not shown: Λ_HS/∨_ for true FS is around −170). Vertical lines indicate the values of *T*_filter_ = −2 (top) and *T*_assign_ = 0.5 (bottom) used throughout the Results. Based on 10 000 simulations of a simple pedigree with unrelated founders and 400 SNPs with MAF 0.3–0.5 and *ε* = 0.005. [Colour figure can be viewed at http://wileyonlinelibrary.com]

Simulated distributions of Λ_PO/∨_ showed a clearer divide between true PO pairs and non‐PO pairs than did Λ_PO/U_ (Fig. 5, left panels). A similar pattern is apparent for FS (middle), and HS (right), although the latter shows less clear separation. Note that when both parents of both individuals are unknown, no HS assignments can be made as it is impossible to distinguish between maternal HS, paternal HS, FA and GP.

The thresholds for an optimal trade‐off between AR and ER will depend on the proportions of different categories of relatives in the sample, which by definition are not known a priori, as well as the number of SNPs and their allele frequencies. Initial explorations showed that for the three different types of simulated data sets and 200 SNPs, results were largely insensitive to varying *T*_Filter_ between −3 and −1, while a value of *T*_Assign_ = + 0.5 gave the best overall trade‐off between AR and ER (Appendix S1, Supporting information). Results will be shown using the same thresholds across all simulations, of *T*_Filter_ = −2 and *T*_Assign_ = + 0.5.

### Parentage assignment

**Figure 6:**
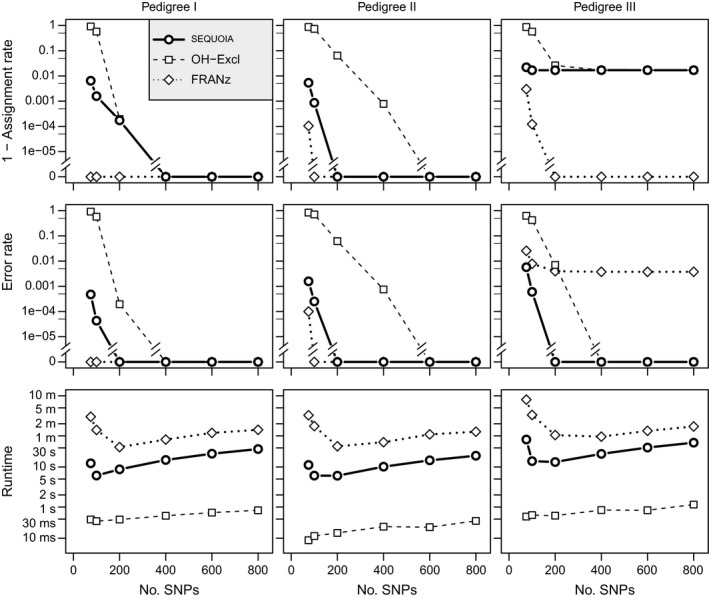
Parent assignment using franz, sequoia (without sibship clustering) or opposite‐homozygosity‐based exclusion (OH‐Excl)in simulated data sets based on three different pedigree structures, with all parental genotypes assumed known. Each point denotes the average over 20 simulations, values are given in Table S4 (Supporting information). Note log scale and broken y‐axes for 1‐AR and ER.

When all individuals are genotyped, assignment rates are high (AR > 99.8% in pedigrees I and II) and error rates low (ER < 0.1%) when at least 100 SNPs are used (Fig. 6, Table S4 in Appendix S1, Supporting information). When using over 400 SNPs, opposite‐homozygosity‐based exclusion (OH‐Exclusion) performs similar to sequoia (ER < 0.1%), in a fraction of the time. franz is somewhat slower than sequoia, but the difference is negligible compared to for example masterbayes (Hadfield *et al*. 2006) which takes many hours for a data set of similar size (C. Berenos, pers. comm.). In Pedigree III, some parents with unknown birth year are never assigned by sequoia or OH‐Exclusion, while franz appears less conservative, resulting in higher AR but also higher ER. Performance of franz in pedigree II was unchanged or worsened when using the option to assist parentage assignment by clustering of full‐siblings (Fig. S6 in Appendix S1, Supporting information).

### Full‐sib clustering

**Figure 7:**
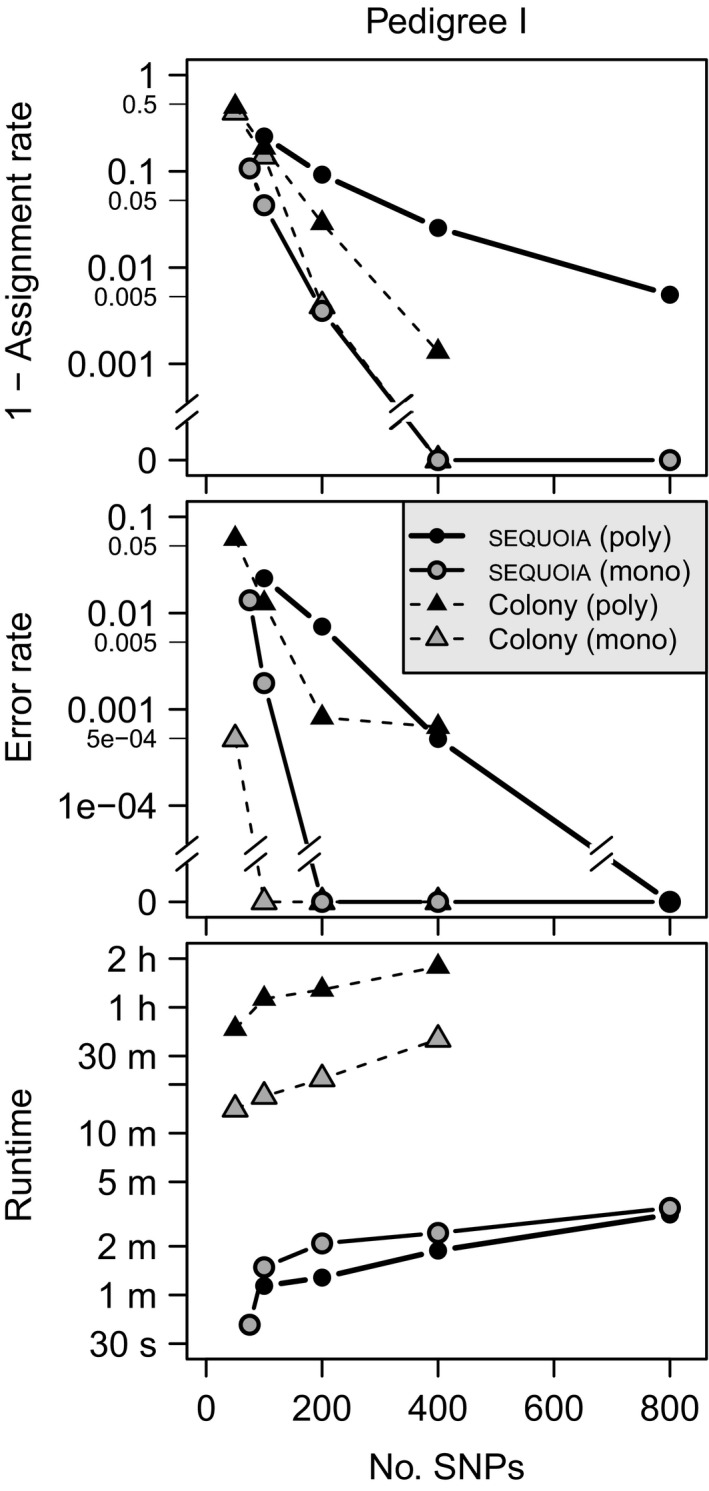
As Fig. 6, for clustering of FS families with no genotyped parents, assuming a polygamous or monogamous breeding system. Averages over 10 replicates (sequoia) or three replicates (colony) were used; colony was not run for 800 SNPs.

Clustering of full‐sib families within a single generation, without any parental genotypes, gave high ARs (>98.4%) and low ER (<0.1%) when at least 200 SNPs were used with sequoia, but ER was consistently higher than for colony (Fig. 7). Even at high marker numbers sequoia erroneously inferred some FS as HS (Fig. S8, see 7 in Appendix S1, Supporting information). Both colony and sequoia performed better when a monogamous breeding system was assumed (grey filled symbols in Fig. 7; Fig. S9 in Appendix S1, Supporting information).

### Combination of parentage assignment and sibship clustering

**Figure 8:**
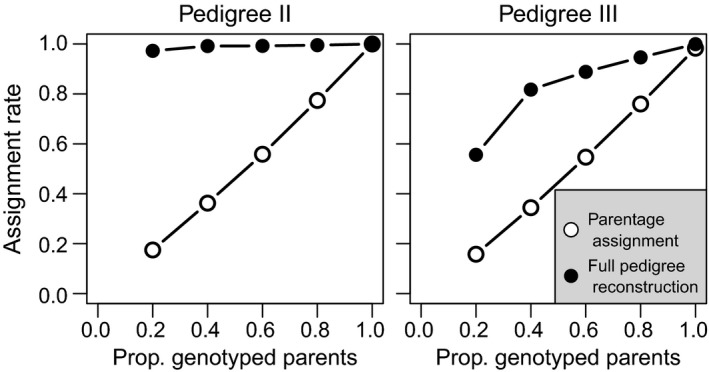
AR of parentage assignment (open circles) is necessarily strongly correlated with the proportion of genotyped parents, but this dependence is much weaker for full pedigree reconstruction (filled circles). Results shown for *L* = 400 SNPs; see Fig. S10 in Appendix S1 (Supporting information) for ER and runtimes.

The combination of parentage assignment, sibship clustering and grandparent assignment resulted in reconstruction of 99% of parent–offspring links in Pedigree II when at least 20% of parental genotypes was treated as known (Fig. 8). When simulating 60% of parental genotypes as known, AR was somewhat lower in Pedigree III at 86%–89% (Table 4), partly because for some identified likely HS it could not be determined whether they were paternal or maternal half‐siblings, or FA. Additionally, when generations overlap and one of a pair of individuals truly is a founder, sequoia cannot differentiate between GG or FA, which would require that one parent is already assigned to each individual. AR for parentage assignment (e.g. franz) is necessarily limited by the number of PO pairs where both are genotyped, while the upper limit for colony is determined by the number of dummy individuals (=number of sibships), to which it does not assign parents.

**Table 4:** Results when 40% of parental genotypes are discarded from the simulated data sets, for a range of marker numbers

Pedigree	SNPs	Assignment rate			Error rate			Computational time^a^		
		franz	sequoia	colony b	franz	sequoia	colony	franz	sequoia	colony
II	75	0.550	0.802	0.951	8.38E−2	4.41E−2	<4.5E−4	01:44	03:45	2:23:00
	100	0.561	0.927	0.955	5.17E−2	1.42E−2	<4.5E−4	01:03	02:54	2:36:00
	200	0.559	0.989	0.958	1.37E−2	1.25E−3	<4.5E−4	00:36	01:53	4:34:00
	400	0.559	0.993		6.50E−4	<5.0E−5		00:42	01:51	
	800	0.564	0.991		<5.0E−5	<5.0E−5		01:13	03:59	
III	75	0.540			1.25E−1			04:12		
	100	0.546	0.725		6.62E−2	2.40E−2		02:11	49:32	
	200	0.554	0.861		1.38E−2	1.48E−3		00:52	27:19	
	400	0.549	0.888		2.98E−3	6.51E−4		00:50	27:38	
	800	0.555	0.894		2.20E−3	7.26E−4		01:28	57:30	

For franz (parentage only) and sequoia, averages over 10 simulations are given, and for colony (polygamous), numbers are extrapolated from running on generations 1 and 5 (founders = 0) for three replicates. Times in minutes: seconds for franz and sequoia, and hours: minutes: seconds for colony.

a On a laptop with a quadcore intel i7 2.3 GHz processor and 8 GB RAM.

b AR = within‐cohort AR – 0.042, to take into account that no grandparents are assigned to the on average 48 + 32 sibships (see data set description).

Error rates for sequoia were low when at least 200 SNPs were used (0.1%–0.3%) and were undetectably low for colony (Table 4) despite both data sets containing closely related parents, which is not explicitly dealt with by this program. Computational time had a minimum around 200 SNPs, increased approximately quadratically with the number of individuals (Fig. S10 in Appendix S1, Supporting information), and was considerably longer for the more complex Pedigree III. A slight increase in ER with increased pedigree size and depth (to 0.26%) and with decreased proportion of genotyped parents (to ER= 0.9%) was observed (Fig. S10 in Appendix S1, Supporting information).

For Pedigree II and 200 SNPs, ER increased and AR decreased approximately exponentially with an increase in simulated genotyping error rate (Fig. S7 in Appendix S1, Supporting information).

### Empirical data sets

**Figure 9:**
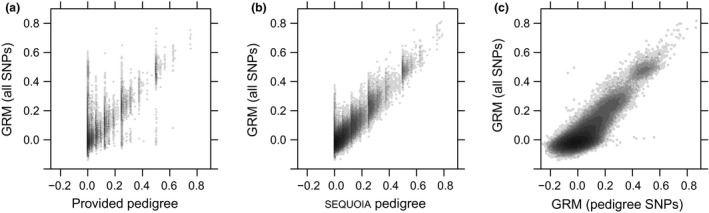
Pairwise relatedness in an empirical red deer data set, as estimated from 40 000 polymorphic SNPs using gcta (y‐axes), and (a) a previous microsatellite‐based pedigree, (b) from the pedigree inferred using sequoia on 440 SNPs with high MAF and in low LD, or (c) from these same 440 SNPs using gcta. *n* denotes the number of pairwise relationships, related to the number of unique individuals *i* as *n* = *i* × (*i* − 1)/2.

As a proxy for the true pedigree relatedness between pairs of individuals, the genomic relatedness *r*_grm_ as estimated by gcta (Yang *et al*. 2011) from all 40 000–50 000 SNPs was used. For each of the three data sets, the relatedness estimated from the sequoia‐reconstructed pedigree (*r*_ped, sequoia_) was more strongly correlated to *r*_grm_ than *r*_ped, FRANz_ (Table 5). Note that correlations differed between the three data sets not only due to the pedigree accuracy, but also due to the proportion of close relatives in the sample (see Fig. 9; if fewer pairs were closely related, the correlation would be lower) and the amount of Mendelian variance, determined by the number and size of chromosomes. Correlations were lowest in the pig data set, amongst others because maternal siblings were often fully nested within paternal sibling groups, which cannot be differentiated from paternal siblings nested within maternal sibling groups when none of the parents are genotyped (see also Discussion). Correlations between *r*_ped, sequoia_ and *r*_ped, provided_ ranged from 0.72 for the red deer data set to 0.87 in the great tits and 0.89 in the pigs.

**Table 5:** Correlations *ρ* between genomic and pedigree relatedness (*r*_grm_ and *r*_ped_, respectively) in three empirical data sets, with three pedigrees each, and rough estimates of pairwise 1 − AR (proportion of pairs with *r*_ped_ − *r*_grm_ < −0.2) and ER (*r*_ped_ − *r*_grm_ > 0.2); proportions are multiplied by 10^5^ to ease comparison

Pedigree	cor(*r*_grm_, *r*_ped_)			*r*_ped_ − *r*_grm_ < −0.2			*r*_ped_ − *r*_grm_ > 0.2		
	Deer	Pig	Tits	Deer	Pig	Tits	Deer	Pig	Tits
Provided^a^	0.66	0.55	0.70	270	1700	110	20	4.1	6.1
Provided^b^	0.45	0.55	0.56	550	1700	110	8.5	4.1	2.9
franz	0.72	0.34	0.53	130	2500	140	0.37	0.13	3.2
sequoia	0.81	0.47	0.64	5.3	2200	71	0.091	4.5	0.096

a Correlation over genotyped individuals present in the pedigree only.

b Assuming that individuals not present in the pedigree are unrelated to all others.

The fraction of pairs with a much higher *r*_ped_ than *r*_grm_ provides a rough estimate of ER, and was consistently lower for sequoia than for franz, and lower than the provided pedigree for the two wild species. The pattern for the fraction of pairs with much higher *r*_grm_ than *r*_ped_ (likely but nonassigned relatives) showed a similar pattern across data sets and pedigrees (Table 5). Note that pairwise AR and ER are not directly comparable to the per‐individual AR and ER reported elsewhere in the Results, as a single erroneous assignment typically results in erroneous *r*_ped_ between multiple pairs (see also Fig. S8 in Appendix S1, Supporting information).

As illustrated for the red deer data set (Fig. 9), *r*_grm_ was more closely correlated to *r*_ped, sequoia_ than to the genomic relatedness estimated from the 440 SNPs used for pedigree reconstruction. This may partly be an artefact of the different average allele frequencies in the two sets of markers, but is probably largely due to Mendelian noise. It suggests that when only a few hundred SNP markers are available, it can be better to estimate quantitative genetic parameters using *r*_ped_ than *r*_grm_.

## Discussion

sequoia enables pedigree inference even with complex mating structures, extensively overlapping generations and inbreeding. Parentage assignment performs very well down to about 100 independent highly informative SNPs, while for subsequent sibship clustering, at least a few hundred SNPs are required. For these marker numbers, false‐positive rates in the simulated data sets are low (<0.1%) and assignment rates high (>99%). As for any software, performance in real data sets will be somewhat lower, but results in three empirical data sets are favourable compared to existing pedigrees and parentage assignment only.

### Comparison to other methods

The main difference in approach between sequoia and most other methods is that a high likelihood solution is found in a handful of iterations, rather than the tens of thousands of iterations typical of MCMC approaches. sequoia's sequential, heuristic method requires a conservative approach to assignments, which results in lower AR than colony under identical conditions. There is also some loss of accuracy, but this can be overcome using a few hundred extra SNPs. When less than approximately 200 independent, high frequency SNPs are available, due to a small genome size or for budgetary reasons, the methods initially developed for a dozen or so microsatellite markers still perform best. For limited marker numbers, Mendelian noise can be substantial, and as a result, the true configuration may not be amongst those with the highest partial likelihood, violating a core assumption underlying sequoia. The true pedigree will typically still have the highest global likelihood, which can be more easily found by MCMC or simulated annealing algorithms such as colony, than by a hill‐climbing algorithm such as sequoia.

### Parentage assignment

When interest is solely in parentage assignment, sequoia performs intermediately between opposite‐homozygosity‐based exclusion and franz (Riester *et al*. 2009). The former performs very well when a large number of markers is available, although allowing for genotyping errors creates room for false‐positive assignments (Strucken *et al*. 2015). franz explicitly deals with genotyping errors and makes use of birth year, death year and gender information, but is less conservative than sequoia when this life‐history information is lacking for some individuals. Note that while franz performs clustering of full‐siblings, it does so only to support parentage assignment, and in a less integrated way than sequoia.

### Sibships

**Figure 10:**
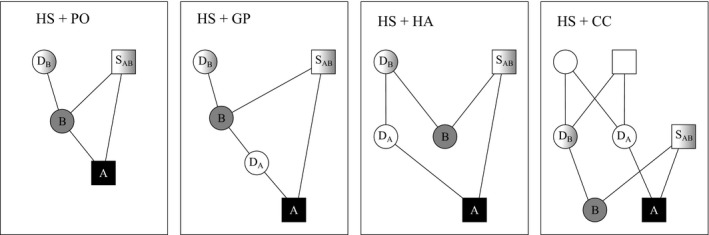
Examples of double relationships between genotyped individuals *A* and *B*, where *D*_*B*_ and *S*_*AB*_ may or may not be genotyped, and *D*_*A*_ is not genotyped. Description and likelihood equations in Methods in Appendix S1 (Supporting information).

It has been observed that likelihood scores tend to favour more complex explanations (Thomas & Hill 2002; Almudevar 2007), resulting in splitting true sibling groups (Almudevar 2007) as well as creation of spurious sibling groups (Anderson & Ng 2016). With sequoia, the number of unrelated pairs spuriously inferred as HS or FS was orders of magnitude lower than nonassignment of true siblings (Fig. S8 in Appendix S1, Supporting information). Nonassignment in Pedigree I was predominantly due to a limited likelihood difference for true full‐siblings to be FS (*r* = 1/2) versus paternal HS and maternally related as HA or CC, for example (*r* = 1/4 + 1/8, Fig. 10). Such configurations might be comparatively common in some species, but very rare in others. A priori estimates of the fraction of pairs in each type of relationship (PO, FS, HS, CC, etc.) are likely to lessen this problem, as implemented in franz (Riester *et al*. 2009) and snppit (Anderson 2012). Assuming a monogamous breeding system could be seen as a special case of this and did indeed improve performance. However, in real data sets, there is typically no a priori certainty about monogamy.

In the empirical red deer data set, sequoia identified many paternal half‐sib links across cohorts, which cannot be identified with per‐cohort sibship clustering using colony. Several birth year cohorts may be analysed together using a sliding‐window approach, but combining the results into a single pedigree is hindered by the presence of erroneous sibship clusters, and the lack of concordance between a sibship's posterior probability and its correctness (Anderson & Ng 2016). More generally, separate reconstruction within each cohort may lead to biologically impossible pedigrees when combining results (Taylor *et al*. 2015) and complicates inclusion of individuals with unknown birth year. In the red deer example, immigrant males were never considered as offspring during paternity assignment, but sequoia identified various paternal links between immigrants.

### Potential caveats

Real‐world data sets are often incomplete and imperfect, especially those for wild populations. For example, birth years may be unknown for many individuals, as was the case for the great tit data set. Nonetheless, the pedigree reconstructed by sequoia showed strong correlation with *r*_grm_, and 81 unique fathers were assigned despite unknown hatching year. In such cases, lists of per‐cohort candidate parents, such as used by masterbayes and colony, may be more convenient than estimating birth years, although great care should be taken to not inadvertently leave out the true parent. Candidate parent lists allow implicit incorporation of data on year of death, when available, which is used explicitly by franz but currently cannot be used by sequoia. Note as well that uncertainty around birth year estimates is currently not accounted for by sequoia, although parent–offspring pairs with impossible or unknown age differences will be flagged.

One general problem with pedigree reconstruction is the differentiation between maternal and paternal relatives. For example for a full‐sib family (*n* ≥ 1) and in absence of parental genotypes, it is impossible to distinguish between maternal and paternal HS. Programs incorporating prior information on observation‐based parents are then preferred, such as colony (Wang 2012). I am not aware of any programs that incorporate data on sex‐linked markers, which would provide an alternative way to differentiate between maternal and paternal relatives.

Currently, no confidence probability is attached to sequoia's assignments, but various methods to estimate these exist (Results and discussion in Appendix S1, Supporting information). For example, one may simulate many SNP data sets according to the observed allele frequencies and inferred pedigree, and count the mismatches between pedigrees reconstructed from simulated data and the initial pedigree, similar to cervus (Marshall *et al*. 1998). To this end, and to investigate the sensitivity of pedigree inference to specific relatedness structures or various other properties of real‐world data sets, including genotyping errors, the r package includes functions to simulate genotypes of unlinked SNPs through any pedigree and to count mismatches between two pedigrees. The sensitivity to various aspects is likely to be data set specific and therefore not explored in detail here.

## Data accessibility

The r package is available on cran (CRAN.R‐project.org/package=sequoia) and includes a pedigree and life‐history file for Pedigree II, a user manual, and the Fortran source code. The red deer SNP data are available from github.com/JiscaH/Manuscripts‐Papers, the great tit data were retrieved from datadryad.org/resource/doi:10.5061/dryad.5t32v (UK part), and the pig data were from http://www.g3journal.org/content/suppl/2012/04/06/2.4.429.DC1.

## Supporting information


**Appendix S1** Supporting Methods and Results.

**Appendix S2** Sequoia R vignette.
