# Supplementary material for: Pedigree reconstruction from SNP data: parentage assignment, sibship clustering and beyond
Source: Mol Ecol Resour. 2017 Apr 6;17(5):1009–24. doi: 10.1111/1755-0998.12665 (PMC6849609; doi:10.1111/1755-0998.12665)
Supplement: Supplementary file 1 — Appendix S1 Supporting Methods and Results. [file MEN-17-1009-s001.pdf]

Supplementary Information for: Pedigree reconstruction from SNP data: Parentage assignment, sibship clustering, and beyond

Jisca Huisman

February 22, 2017

Contents

S1 Supplementary Methods **2**
S1.1 Genotyping error model . . . 2
S1.2 Likelihood equations . . . 2
S1.2.1 Likelihoods for close inbreeding and double relatives . . . 4
S1.2.2 Inbreeding within sibship cluster . . . 6
S1.2.3 Interconnected sibships . . . 6
S1.3 Differentiating between types of second degree relatives . . . 6
S1.4 Parentage assignment . . . 7
S1.5 Age-difference based priors . . . 8
S1.5.1 Absence of age or sex information . . . 9
S1.6 Sibship clustering . . . 9
S1.7 Simulation of SNP data . . . 10

S2 Supplementary Results and Discussion **10**
S2.1 Threshold for assignment . . . 10
S2.2 Algorithm order . . . 11
S2.3 Assignment confidence . . . 12
S2.4 Additional tables and figures . . . 13

S1 Supplementary Methods

S1.1 Genotyping error model

To account for genotyping errors, I distinguish between the observed genotype X, and the actual genotype x. I assume the genotyping error rate ϵ is constant across loci, that errors occur independently of each other, and that there are no (heritable) mutations.

In the default error structure used, the chance of observing a true minor homozygote as major homozygote, or vice versa, is assumed negligible (Table S1), based on own observations when scoring SNP array data in Illumina's GenomeStudio. True heterozygotes are observed as either homozygote with probability $\epsilon/2$, and true homozygotes are observed as heterozygous with probability ϵ, i.e. a allelic-dropout-like error structure. Different error structures can be easily implemented by changing the analogue of Table S1 in the source code.

Table S1: Default probabilities used of observing genotype X, conditional on actual genotype x.

	X		
x	0	1	2
0	1-ϵ	ϵ	0
1	$\epsilon/2$	1-ϵ	$\epsilon/2$
2	0	ϵ	1-ϵ

The probability that a given observed genotype is erroneous depends slightly on the allele frequency, similar to in e.g. Wang (2004). Intuitively, the reason is that an observed homozygote for a very rare allele may be less likely to be an actual homozygote (with probability q^2) than to be an actual heterozygote ($\epsilon/2 \cdot 2q(1-q)$). Note however that in practice, q will typically exceed ϵ by several orders of magnitude.

More formally, the joint distribution P(Observed, Actual) is a function of the frequencies of the actual genotypes, which are assumed to be according to HWE, and the conditional probabilities P(Actual | Observed) are calculated as

$$P(\text{Actual}|\text{Observed}) = \frac{P(\text{Actual, Observed})}{P(\text{Observed})} = \frac{P(\text{Observed}|\text{Actual})P(\text{Actual})}{\sum_{x=0}^{2} P(\text{Observed}|\text{Actual}=x)}, \tag{S1}$$

where P(Actual) assumes HWE when both parents of the focal individual are unknown, and is otherwise dependent on the parental genotype(s) (see Equation 3). The error model used assumes a slightly inflated number of observed heterozygotes (of $2q(1-q)+\epsilon$), which will bias the estimated allele frequencies $\hat{q}$ upwards by $1/2\epsilon(1-2q)$. This bias is corrected for, but is negligible for typical error rates ($\epsilon = 0.005 - 0.01$, (Anderson & Garza, 2006)) and the most informative allele frequencies (q =0.3–0.5).

Note that null alleles are currently not explicitly incorporated. They are much less of a problem than with microsatellite markers, but occasionally a third allele may occur at a SNP locus, which binds to neither probe of the SNP array. Heterozygotes for such an allele may either be scored as homozygote, or not at all. Typically, however, such tri-allelic SNPs are excluded during quality control, as they either have low call rate, or show considerable deviation from HWE.

S1.2 Likelihood equations

The general approach of the likelihood equations is explained in the Methods. Here, as an example, all pairwise likelihood equations are shown for a pair of individuals A and B, for a maternal focal relationship, and B being older than A (e.g. the question whether B is the mother of A). For paternal focal relationships, one can simply swap the interpretation of the parental symbols below, currently meaning D_A for A's mother, and S_A for A's father.

Each equation can be generalised to the situation where either or both A and B are sibships, rather than individuals, in which case one multiplies over all members of the sibship(s), analogous to Equation 4.

H_0: **Unrelated** Firstly, the likelihood under the hypothesis that the pair is conditionally unrelated is

$$\mathcal{L}(\mathrm{U}|A, B, \ldots) = \mathcal{L}(A, D_A, S_A)\mathcal{L}(B, D_B, S_B) \tag{S2}$$

where $\mathcal{L}(A, D_A, S_A)$ is defined in Equation 2, and $\ldots$ denotes the parents of A and B when known.

H_1–H_2: **First degree relatives** The first alternative relationship (H_1) considered is parent-offspring (PO), with say B being the candidate mother of A

$$\mathcal{L}(\mathrm{PO}|A, B, \ldots) = \prod_{l=1}^{L} \prod_{l} \sum_{y} \sum_{z} \sum_{v} \sum_{w} P_{M\epsilon}(A = X|B = y, S_A = z) P_\epsilon(B = Y|B = y, \epsilon) P_P(S_A = z) \times \\ P_M(B = y|D_B = v, S_B = w) P_P(D_B = v) P_P(S_B = w) \ , \tag{S3}$$

dropping subscripts l for brevity, and using the shorthand

$$P_{M\epsilon}(A = X|B = y, S_A = z) = \sum_{x} P_\epsilon(A = X|A = x, \epsilon) P_M(A = x|B = y, S_A = z) \tag{S4}$$

If A and B do not have a different mother or a different father assigned, secondly (H_2) the likelihood of being full siblings (FS) is calculated,

$$\mathcal{L}(\mathrm{FS}|A, B, \ldots) = \prod_{l=1}^{L} \prod_{l} \sum_{u} \sum_{z} P_{M\epsilon}(A = X|D_{AB} = u, S_{AB} = z) \times \\ P_{M\epsilon}(B = Y|D_{AB} = u, S_{AB} = z) P_P(D_{AB} = u) P_P(S_{AB} = z) \ , \tag{S5}$$

where D_{AB} and S_{AB} are the shared parents of A and B.

H_3–H_5: **Second degree relatives** The likelihood that A and B are maternal half-siblings is given by

$$\mathcal{L}(\mathrm{HS}|A, B, \ldots) = \prod_{l=1}^{L} \prod_{l} \sum_{u} \sum_{z} \sum_{w} P_{M\epsilon}(A = X|D_{AB} = u, S_A = z) \times \\ P_{M\epsilon}(B = Y|D_{AB} = u, S_B = w) P_P(D_{AB} = u) P_P(S_A = z) P_P(S_B = w) \ , \tag{S6}$$

and that they are grandparent and grand-offspring by (here via D_A; via S_A is considered too)

$$\mathcal{L}(\mathrm{GG}|A, B, \ldots) = \prod_{l=1}^{L} \prod_{l} \sum_{u} \sum_{y} \sum_{z} \sum_{v} \sum_{w} \sum_{t} P_{M\epsilon}(A = X|D_A = u, S_A = z) P_P(S_A = z) \times \\ P_M(D_A = u|B = y, MGF_A = t) P_M(B = y|D_B = v, S_B = w) \times \\ P_P(D_B = v) P_P(S_B = v) P_P(MGF_A = t) P_{P^*}(D_A = u) \ , \tag{S7}$$

where MGF_A is the maternal grandfather of A, and $P_{P^*}(D_A = u) = P_\epsilon(D_A = U|D_A = u)$ for D_A known and genotyped, $P_{P^*}(D_A = u) = 1$ for D_A unknown, and when D_A is a dummy parent calculated from $\mathcal{L}(\boldsymbol{A})$ without the contributions of either grandparent or A.

The fifth alternative is full avuncular (H_5), i.e. either parent of A (here D_A) is a full sibling of B,

$$\mathcal{L}(\mathrm{FA}|A, B, \ldots) = \prod_{l=1}^{L} \prod_{l} \sum_{u} \sum_{z} \sum_{v} \sum_{w} P_{M\epsilon}(A = X|D_B = u, S_A = z) P_{M\epsilon}(B = Y|D_B = v, S_B = z) \times \\ P_M(D_A = u|D_B = v, S_B = z) P_P(S_A = z) P_P(D_B = v) P_P(S_B = w) P_{P^*}(D_A = u), \tag{S8}$$

where S_A also might be a FS of B, or either parent of B a FS of A.

As mentioned in the Introduction, $\mathcal{L}$(HS | A, B) = $\mathcal{L}$(GG | A, B) = $\mathcal{L}$(FA | A, B) when neither A nor B has a (dummy)parent assigned. Distinction between these three relationship types can be made when either parent of B is known (Appendix C), or when the age difference between A and B excludes some of the configurations (Appendix D).

H_6: **Third degree relatives** Lastly, A and B may be third degree relatives. These are considered to prevent false positive assignments, as some third degree relatives may have a higher likelihood to be second degree relatives than unrelated - but will have an even higher expected likelihood to be third degree relatives. Assigning third degree relatives is not attempted, as the distinction with fourth degree relatives is difficult. Moreover, even if B were known to be a great-grandparent of A, it would be unclear which of the 8 great-grandparents of A it was, without knowledge on first and second degree relatives of A and B.

The likelihood to be third degree relatives is taken as the most likely scenario of half-avuncular (HA), great-grand-parental (GGG), or full first cousins (CC):

$$\begin{aligned}
\mathcal{L}(\text{HA}|A,B) = \prod_l \sum_u \sum_z \sum_v \sum_w \sum_t & P_{M\epsilon}(A=X|D_A=u, S_A=z) P_{M\epsilon}(B=Y|D_B=v, S_B=w) \times \\
& P_M(D_A=u|D_B=v, MGF_A=t) P_P(S_A=z) P_P(D_B=v) P_P(S_B=w) \times \\
& P_P(MGF_A=t) P_{P*}(D_A=u) \qquad \text{(S9)} \\
\mathcal{L}(\text{GGG}|A,B) = \prod_l \sum_y \sum_u \sum_z \sum_v \sum_w \sum_s \sum_t & P_{M\epsilon}(A=X|D_A=u, S_A=z) P_P(S_A=z) \times \\
& P_M(D_A=u|s, MGF_A=t) P_{Mh}(s|B=y, q_l) P_M(B=y|D_B=v, S_B=w) \times \\
& P_P(D_B=v) P_P(S_B=v) P_P(MGF_A=t) P_{P*}(D_A=u) \ , \qquad \text{(S10)} \\
\mathcal{L}(\text{CC}|A,B) = \prod_l \sum_u \sum_z \sum_v \sum_w \sum_s \sum_t & P_{M\epsilon}(A=X|D_A=u, S_A=z) P_P(S_A=z) \times \\
& P_{M\epsilon}(B=Y|D_B=v, S_B=w) P_P(S_B=w) P_M(D_A=u|MGF_{AB}=t, MGM_{AB}=s) \times \\
& P_M(D_B=v|MGF_{AB}=t, MGM_{AB}=s) P_P(MGF_{AB}=t) P_P(MGM_{AB}=s), \\
& \qquad \text{(S11)}
\end{aligned}$$

where $P_{Mh}(s|B=y, q_l)$ is the inheritance probability from a single parent (here B), and MGF_A and MGM_A are the maternal grandmother and maternal grandfather of A, respectively. Under HA, when the focal hypothesis is that A and B are relatives of type k, we consider the possibilities that parent k of A is a paternal or maternal half-sibling of B, or that parent k B is a paternal or maternal half-sibling of A.

It can be shown that similar to 2nd degree relatives, all 3rd degree relatives have the same likelihood function when not conditioning on any parental or sibling genotypes. Therefore, full great-uncle – great-nephew pairs, which would require summation over four (unobserved) relatives, are currently not explicitly considered, as they either have a similar likelihood as HA or GGG, or one of the 'intermediate' individuals is known making A and B conditionally unrelated.

Note that although there are up to 6 or 7 summations in each likelihood equation, many short-cuts can be taken by the use of look-up tables. Moreover, as there are only 3 different possible states per individual, this constitutes only $3^6 = 729$ different joined states - identical to the number of possible states for a trio on a microsatellite locus with 9 alleles, a very typical number.

S1.2.1 Likelihoods for close inbreeding and double relatives

The explicit consideration of inbred configurations is not required to detect those, as they will typically come about as by-products of parentage assignment and sibship clustering. However,

when exploring the alternative hypothetical relationships, complex relationships (Figure 10) need to be considered to avoid erroneous assignments. For example, if a mother and daughter mate with the same male (as common in a.o. red deer, (Stopher *et al.*, 2012)), their offspring are related by $1/4 + 1/8 = 0.375$ (HS + HA) and can easily be mis-identified as full siblings if HS + HA is not explicitly considered. Erroneous assignment as a FS pair can have considerable downstream consequences, by providing an erroneous 'core' from which a sibship may grow.

When the focal individual is the result of a parent-offspring mating (HS + PO in Figure 10), the assumption that the opposite-sex parent is a random draw from the population is severely violated (${}^{h}P_P$ defined in Methods, below Equation 3). When considering say candidate mother B for focal individual A (following the labelling in Figure 10), we consider the possibility that A and B may share the same father S_{AB},

$$\begin{aligned}\mathcal{L}(\text{PO + HS}|A, B) = \prod_{l=1}^{L} \prod_{l} \sum_{y} \sum_{z} \sum_{v} & P_{M\epsilon}(A = X|B = y, S_{AB} = z) P_P(S_{AB} = z) \times \\ & P_M(B = y|S_{AB} = z, D_B = v) P_\epsilon(B = Y|B = y, \epsilon) P_P(D_B = v) \ ,\end{aligned} \tag{S12}$$

as well as the alternative configuration, illustrated as S_{AB} being a candidate father, and now assuming $D_B = A$ non-genotyped,

$$\begin{aligned}\mathcal{L}(\text{PO + GP}|A, S_{AB}) = \prod_{l=1}^{L} \prod_{l} \sum_{y} \sum_{z} \sum_{v} & P_{M\epsilon}(A = X|D_A = y, S_{AB} = z) P_P(S_{AB} = z) \times \\ & P_M(D_A = y|S_{AB} = z, D_{D_A} = v) P_\epsilon(S_{AB} = Y|S_{AB} = y, \epsilon) \ .\end{aligned} \tag{S13}$$

The maximum of $\mathcal{L}(\text{PO})$, $\mathcal{L}(\text{PO + HS})$ and $\mathcal{L}(\text{PO + GP})$ is used in comparison with the likelihoods of the alternative scenarios.

When during sibship clustering a pair has a higher likelihood to be FS than any of the other standard relationships, we additionally consider whether they may be HS plus a different type of second degree relationship, or HS plus a third degree relationship. For example, when the parents D_A and D_B of half-siblings A and B are PO, then A and B are also HA, and

$$\begin{aligned}\mathcal{L}(\text{HS + HA}|A, B) = \prod_{l=1}^{L} \prod_{l} \sum_{z} \sum_{v} \sum_{w} \sum_{u} & P_{M\epsilon}(A = X|D_A = w, S_{AB} = z) P_P(S_{AB} = z) \times \\ P_{M\epsilon}(B = Y|D_B = v, S_{AB} = z) & P_M(D_A = w|D_B = v, S_{D_A} = u) P_P(D_B = v) P_P(S_{D_A} = u) \ ,\end{aligned} \tag{S14}$$

assuming D_A is non-genotyped. If D_A and D_B are FS than A and B are HS plus first cousins,

$$\begin{aligned}\mathcal{L}(\text{HS + CC}|A, B) = \prod_{l=1}^{L} \prod_{l} \sum_{z} \sum_{v} \sum_{w} \sum_{u} \sum_{v} \sum_{t} & P_{M\epsilon}(A = X|D_A = w, S_{AB} = z) P_P(S_{AB} = z) \times \\ & P_{M\epsilon}(B = Y|D_B = v, S_{AB} = z) P_M(D_A = w|D_{D_A D_B} = u, S_{D_A D_B} = v) \times \\ & P_M(D_B = v|D_{D_A D_B} = u, S_{D_A D_B} = v) P_P(D_{D_A D_B} = u) P_P(S_{D_A D_B} = v) \ ,\end{aligned} \tag{S15}$$

assuming both D_A and D_B are non-genotyped. If $\mathcal{L}(\text{HS + HA})$ or $\mathcal{L}(\text{HS + CC})$ exceeds $\mathcal{L}(\text{FS})$ than $\mathcal{L}(\text{FS})$ is set to missing, as in absence of any assigned parents it cannot be determined whether A and B are paternal HS with related mothers, or maternal HS with related fathers.

The modular structure of the source code allows additional types of relationships to be added quite easily. This may be required if they are common in the population of interest, and otherwise provide a large source of false positives. Double grandparents cannot be considered explicitly, as their likelihood is indistinguishable from PO.

S1.2.2 Inbreeding within sibship cluster

Within a sibship, a grandparent may also be an opposite-sex parent of one of the members, or one sibship member may be the opposite-sex parent of another member. To incorporate these possibilities, Equation 4 is generalised to

$$\mathcal{L}(\boldsymbol{A}) = \prod_l \sum_x \sum_v \sum_w P_M(D_{\boldsymbol{A}} = x | GM_{\boldsymbol{A}} = v, GF_{\boldsymbol{A}} = w) P_P(GM_{\boldsymbol{A}} = v) P_{P'}(GF_{\boldsymbol{A}} = w) \times$$
$$\prod_{i=1}^{n_A} I(S_i = GF_{\boldsymbol{A}}) \prod_{j=1}^{m_{A,i}} P_{M\epsilon}(A_{i,j} = Z | D_{\boldsymbol{A}} = x, GF_{\boldsymbol{A}} = w) I(S_i \in \boldsymbol{A}) \times$$
$$\sum_u P_{M\epsilon}(S_i = y_i | D_A = x, S_{Si} = u) P_P(S_{Si} = u) P_{M\epsilon} \prod_{j=1}^{m_{A,i}} (A_{i,j} = Z | D_{\boldsymbol{A}} = x, S_i = y_i) \times$$
$$I(S_i \neq GF_{\boldsymbol{A}}) I(S_i \notin \boldsymbol{A}) \sum_{y_i} P_P(S_i = y_i) \prod_{j=1}^{m_{A,i}} P_{M\epsilon}(A_{i,j} = Z | D_{\boldsymbol{A}} = x, S_i = y_i) \;, \qquad \text{(S16)}$$

where $P_{P'}(GF_{\boldsymbol{A}} = w)$ is calculated without the contribution of its shared offspring with D_A, and I are indicator variables taking the value 1 when true and 0 when false.

S1.2.3 Interconnected sibships

When considering various hypothesised relationships between sibship $\boldsymbol{A}$ and individual B, or between sibships $\boldsymbol{A}$ and $\boldsymbol{B}$, likelihood calculations are mostly performed over the sibship cluster itself, and all sibships directly linked to it (e.g. for a maternal sibship, all paternal sibships of the males with whom the sibship mother mated). This is especially useful when there are multiple opposite-sex dummy parents, as using ${}^dP_P(S_1 = y_1|x = 0)$ and ${}^dP_P(S_2 = y_2|x = 0)$, will give different results from using $\sum_{x'} {}^dP_P(S_i = y_i|D_A = x') {}^dP_P(D_A = x')$ for $i = 1, 2$. Especially when $\boldsymbol{A}$ is small may ${}^dP_P(S_1)$ and ${}^dP_P(S_2)$ depend strongly on each other; for example, if at a locus A_1 and A_2 are both heterozygous, if $D_{\boldsymbol{A}} = 2$, most likely $S_1 = S_2 = 0$, while when $D_{\boldsymbol{A}} = 0$ most likely $S_1 = S_2 = 2$.

In some simpler scenarios, such as addition of a half-sibling with no current parents, changes in the connected sibships are presumed negligible. The more distantly, indirectly connected sibships are always conditioned upon (as assigned in the current pedigree), rather than incorporated in the likelihood. The latter approximation is necessary as the number of interconnected sibship may become very large, and calculations over such large webs are computational intensive, while their contribution to changes in the likelihood is much smaller than the contributions of the focal and directly-connected sibships.

To enable comparison of likelihoods calculated under the different hypotheses, only the likelihood over the focal individuals should be returned. Therefore, in parallel the likelihood over all individuals *except* $\boldsymbol{A}$ and the other focal individual or sibship is calculated, and the required likelihood is taken as the difference between the two.

S1.3 Differentiating between types of second degree relatives

A long standing problem in pedigree reconstruction is the differentiation between half-sibling (HS), grandparent–grand-offspring (GG) and avuncular (FA) pairs (Figure S1) (see e.g. Epstein *et al.*, 2000), which all have an pedigree relatedness of $r = 0.25$. One remedy is to make use of the age difference of the pair (Appendix D), but this provides no conclusive distinction in species where the maximum reproductive lifespan is several times longer than the minimum generation time. Therefore, I (additionally) condition on the genotype of the (dummy)parents of the pair.

It is common practice to condition on the maternal genotype when inferring paternities (e.g. Marshall *et al.*, 1998), because if an heterozygous individual has a major homozygote as mother, it must have inherited the minor

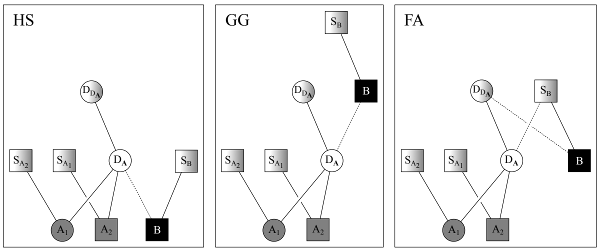

Figure S1: The three types of second degree relationships possible between individual B and sibship $\boldsymbol{A}$ with siblings A_1 and A_2. The genotypes of the fathers (S_A, S_B and S_{AB}) are assumed unknown (denoted by grey square boxes), and the genotypes of the mothers (D_{Ai} and D_B) may or may not be known.

allele from its father (bare genotyping errors). Similarly, this approach has been used to distinguish between full sibs, half sibs and unrelated individuals within a single cohort (Wang, 2004). To our knowledge, however, such an approach has not been widely used to distinguish between types of second degree relatives (but see Anderson & Garza (2006)).

To illustrate, presume $B = 1$ (heterozygous) and $D_B = 2$ (homozygous for the rare allele, as in the right column in Figure S2). Under scenario HS (see Figure S1), S_A ($=S_{AB}$) must be a carrier of the common allele, and

$$\begin{aligned} &P(S_A = 0|\text{HS}, B = 1, D_B = 2) \\ &\qquad = \frac{q^2}{q^2 + 2q(1-q)} = \frac{q}{2-q} \\ &P(S_A = 1|\text{HS}, B = 1, D_B = 2) \\ &\qquad = \frac{2q(1-q)}{q^2 + 2q(1-q)} = \frac{2(1-q)}{2-q} \\ &P(S_A = 2|\text{HS}, B = 1, D_B = 2) = 0, \end{aligned} \tag{S17}$$

while under scenario GG, S_A only depends on B, and is conditionally independent of D_B, and the probabilities are given by:

$$\begin{aligned} P(S_A = 0|\text{GG}, B = 1) &= \frac{1-q}{2} \\ P(S_A = 1|\text{GG}, B = 1) &= \frac{1}{2} \\ P(S_A = 2|\text{GG}, B = 1) &= \frac{q}{2}. \end{aligned} \tag{S18}$$

Lastly, under scenario FA, S_A only depends on D_B, and is conditionally independent of B:

$$\begin{aligned} P(S_A = 0|\text{FA}, D_B = 2) &= 0 \\ P(S_A = 1|\text{FA}, D_B = 2) &= 1 - q \\ P(S_A = 2|\text{FA}, D_B = 2) &= q. \end{aligned} \tag{S19}$$

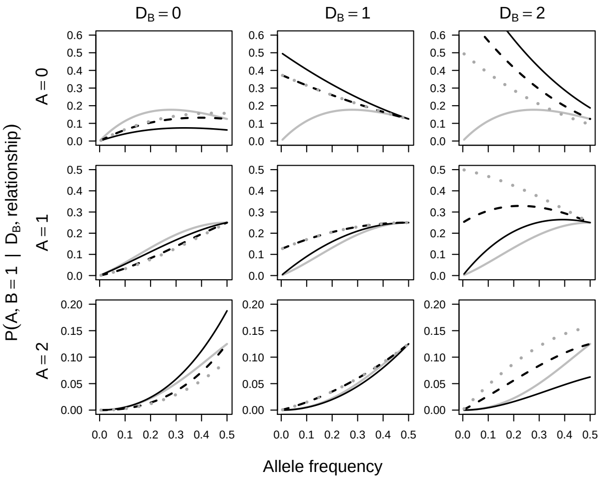

Figure S2: As Figure 3, illustrating how knowledge of the genotype of B's parent D_B (columns) allows differentiation between HS (solid black lines), GG (dashed black) or FA (dotted grey), while these are indistinguishable when D_B is unknown (solid grey lines). Formulae are given in Equations S17– S19 for a single member A_i.

These different probabilities for the possible genotypes of the unobserved S_A, result in different probabilities for the observed genotypes A and B, for all possible genotypes of D_B, as illustrated in Figure S2, and thus different likelihoods for three alternative relationships. Knowledge on D_A does not help in the differentiation, as it does not affect either the probability that S_A inherits an allele from B, nor the reverse probability. However, when generations overlap or the age difference between A and B is unknown, both D_A and D_B are required, as it cannot be determined with certainty whether B might be a full aunt or uncle of A, or instead A an aunt/uncle of B.

S1.4 Parentage assignment

Parentage assignment is done by calculating the pairwise likelihood between the focal individual A and candidate parent B conditional on the parent(s) in the current pedigree, S_A and D_A ($H_{1,\,1,\,0-6}$), as well as under the hypotheses that S_A is unrelated ($H_{0,\,1,\,0-6}$) or D_A is unrelated ($H_{1,\,0,\,0-6}$) (columns in Table S2). To these pairwise likelihoods over A and B the likelihoods of D_A and S_A are added, to obtain the total likelihood over all 2–4 individuals involved. Similarly, the pairwise likelihoods over A and current parent S_A are calculated under

Candidate parent (B)

	Currently assigned father (S_A)						
	**PO**	**FS**	**HS**	**GG**	**FA**	**HA**	**U**
**PO**	$B + S_A$	B	B	B	B	B	B
**FS**	$D_A + S_A$						D_A
**HS**	$D_A + S_A$						D_A
**GG**	$D_A + S_A$						D_A
**FA**	$D_A + S_A$						D_A
**HA**	$D_A + S_A$						D_A
**U**	$D_A + S_A$	D_A	D_A	D_A	D_A	D_A	D_A

Candidate parent (B)

	Currently assigned mother (D_A)						
	**PO**	**FS**	**HS**	**GG**	**FA**	**HA**	**U**
**PO**	N/A	$B + S_A$	$B + S_A$	$B + S_A$	$B + S_A$	$B + S_A$	$B + S_A$
**FS**	$D_A + S_A$						S_A
**HS**	$D_A + S_A$						S_A
**GG**	$D_A + S_A$						S_A
**FA**	$D_A + S_A$						S_A
**HA**	$D_A + S_A$						S_A
**U**	$D_A + S_A$	S_A	S_A	S_A	S_A	S_A	S_A

Table S2: Scheme of quartet relationships considered between a focal individual A, a candidate parent (B, here assumed female) (rows), its previously assigned father S_A (top) and mother D_A (bottom). Bold abbreviations as in Table 1. Values in middle cells indicate which individuals will be assigned as parents when that particular combination of pairwise relationships has the highest likelihood; blank cells are not considered. Note that consideration of candidate mother B may result in joint assignment with current father S_A (when $\mathcal{L}$(PO + PO) in upper matrix exceeds all others), but also in loss of a currently assigned parent.

the condition of B being the parent ($H_{0-6,\,,1,\,1}$, top row in Table S2) or unrelated ($H_{0-6,\,1,\,0}$, bottom row), and the likelihoods of B and D_A addded, and analogously for D_A.

Calculation of the likelihoods under all $2 \times 7 \times 7 = 98$ possible quartet scenarios appears redundant; if for example candidate B truly were a grandmother of A, and S_A truly a full sibling, the likelihoods under the hypothesis (U + FS) and/or (GG + U) would still exceed the likelihoods under the hypothesis (PO + FS) and (GG + PO), and the correct assignment made. During initial parentage assignment, A, B, D_A and S_A are always real genotyped individuals. During later parentage assignment, D_A and S_A may also be dummy parents. In that case, when scaling the pairwise likelihood between A and B to the joined likelihood over all individuals involved, the contribution of A to say D_A's sibship is first divided out, to avoid double counting.

Assignment of dummy parents to A, i.e. sibship clustering, is performed in a separate step from assignment of real parents. In contrast, when assigning grandparents to sibship clusters $\boldsymbol{A}$, this step considers jointly all possible candidates, both genotyped and dummy individuals.

S1.5 Age-difference based priors

The age difference between individuals can be very informative in pedigree reconstruction, as grandparents will on average always be older than siblings, and in many species the two age distributions may show little overlap. Ideally, the effect of age difference on relationships is estimated jointly with the effect of genotypes in a Bayesian MCMC framework (as e.g. in MasterBayes, (Hadfield *et al.*, 2006)). However, this approach can be very time-consuming when the numbers of individuals and markers are large. As an heuristic approximation, we assume that the distribution of maternal and paternal ages amongst assigned parents is identical to that amongst non-genotyped parents. When a sufficient number of individuals has been assigned a sampled parents (by default a threshold of 25% is used), it is possible to estimate from these parental age distributions the empirical age-difference distributions for maternal and paternal siblings, maternal grandmothers, paternal grandfathers, paternal grandmothers and maternal grandfathers, and avuncular pairs. After extending the tails of these distributions, to allow for biologically plausible but unobserved values, and optional smoothing, use of these age-based priors aids in the distinction between various relationships in cases where the genetic data is inconclusive. Implicitly it is assumed that the age distribution and distribution of female and male age at reproduction remain approximately constant throughout the sampling period. These assumptions can be relaxed in future versions if needed, by using birth years rather than, or in addition to, age differences. The age prior appears robust against variation in sampling number and sampling proportion between years, but this has not been rigorously tested.

We account for the fact that the distribution of absolute age differences within the sample is non-uniform, due to the finite time period in which samples are necessarily collected. For example, in a 10 year study period, sampling each individual at birth, many more sampled

individuals will have been born 2 years apart than 10 years apart. We first calculate the proportion of all pairs of individuals which were born t years apart, $P_{U,t}$, with $\sum_{t=0}^{t_{max}} P_{U,t} = 1$. We similarly calculate the number of mother-offspring pairs born t years apart, as a fraction $P_{D,t}$ of the total number of assigned mother-offspring pairs, as well as for father-offspring pairs, $P_{S,t}$, maternal siblings $P_{MS,t}$ and paternal siblings $P_{PS,t}$. From this we calculate the age-difference probability ratio

$$APR_{\cdot,t} = \frac{P_{\cdot,t}}{P_{U,t}} \ , \tag{S20}$$

which is stored in a user-editable text file, rounded to 3 decimal places.

The age distribution of maternal grandmother - grandoffspring $P_{MGM,t}$ is obtained as

$$\begin{aligned} X_{MGM,u,v} &= \sum_{u=0}^{t_{max}} \sum_{v=0}^{t_{max}} P_{D,u} P_{D,v} \\ P_{MGM,t} &= \sum_{u+v=t} X_{MGM,u,v} \ , \end{aligned} \tag{S21}$$

and analogous for paternal grandfathers (PGF) and maternal grandfathers (MGF) / paternal grandmothers (PGM). As a parsimoneous approximation, we assume the same age distribution for paternal and maternal aunts and uncles, as well as for full and half aunts and uncles. It is calculated from the grandparental and parental age distributions as

$$\begin{aligned} X_{AU,u,v} = \frac{1}{4} \sum_{u=0}^{t_{max}} \sum_{v=0}^{t_{max}} & P_{D,t} P_{MGM,t} + \\ & P_{D,t} P_{PGM,t} + P_{S,t} P_{MGF,t} + P_{S,t} P_{PGF,t} \\ P_{AU,t} = \sum_{u+v=t} & X_{AU,u,v} \ . \end{aligned} \tag{S22}$$

S1.5.1 Absence of age or sex information

During parentage assignment, all opposite-sex pairs of candidate parents for each individual are considered. When the sex of one candidate parent (say B) is unknown, it is considered in pairwise combinations with all other candidate parents, of both sexes. Once such an individual is assigned as father or mother, it is subsequently treated as a male respectively female. Note that the likelihood for B to be a parent instead of the other parent is considered as well as the likelihood that both are parents, and a sex only assumed for B once it is assigned as part of a parent pair.

When the age difference between the candidate parent-offspring pair A and B is unknown, an assignment can often still be made. When individual A already has a parent D_A assigned, of different sex than B, the likelihood of B being a joined parent of A will differ from B being an offspring of A. Alternatively, if A has unknown birth year, and has no parents but does have offspring, B must be at least 2 years (time-units) older than A's offspring to be assigned as A's parent.

S1.6 Sibship clustering

Clustering is performed by, for each candidate pair in turn, either using the pair to found a new sibship, when neither individual had yet been assigned to a sibship of type k (maternal or paternal); adding it to an existing sibship, when one of the pair was already assigned to a sibship of type k in an earlier step, or using it to merge two existing sibships, when the pair members were previously assigned to different sibships of the same type. Each step is followed through only when $\mathcal{L}(\text{FS})$ or $\mathcal{L}(\text{HS})$, calculated over the pair and all putative siblings, exceeds the likelihoods under all other relationships ($\max(\Lambda_{HS/\vee}, \Lambda_{FS/\vee}) > T_{assign}$).

After clustering of all candidate pairs, all sibships of the same type are considered for merging, to minimise erroneous splitting of true sibships. Note that the relationship between sibships $\boldsymbol{A}$ and $\boldsymbol{B}$ can be expressed as the relationship between $A_1, A_2, \ldots A_n$ and dummy parent $D_{\boldsymbol{B}}$, so that the considered relationships become identical to the relationships considered for pairs of individuals, as listed in Table 1.

Subsequently, all individuals who lack a parent of type k are considered for addition to each sibship of type k, to ensure that individuals who are ambivalent with respect to their relationship towards other lone individuals, may get clustered due to the combined 'evidence' provided by those in an existing sibship.

S1.7 Simulation of SNP data

To simulate SNP genotypes, each pedigree was split into generations, where those in generation 1 had neither parent known, those in generation 2 had either both parents in generation 1, or one parent in generation 1 and one unknown parent, and those in generation g had parents in generations $< g$, and possibly an unknown parent. Founder genotypes ($i_l = 0$, 1 or 2) were simulated by drawing twice from a binomial distribution, with probabilities equal to the frequency q_l of the reference allele, in turn drawn from a uniform distribution between 0.3 and 0.5 (mimicking a selected subset of highly informative SNPs). Loci were simulated as unlinked, and in linkage equilibrium amongst founders. For subsequent generations, the parental inherited alleles were drawn from a binomial distribution with probability equal to half the parental genotype if known, and probability q_l otherwise. Data was made more realistic by setting 0.5% of individual locus genotypes as missing, and replacing 0.1% of genotypes by a random genotype, which may or may not be identical to the original one. This is a low error rate compared to some estimates (e.g. 0.5% per gene copy, Anderson & Garza (2006)), but a realistic one after stringent quality control even in non-model species (e.g. 0.05%, Hoffman *et al.* (2012)).

The function to simulate genotype data from a known pedigree is included in the R package.

S2 Supplementary Results and Discussion

S2.1 Threshold for assignment

As stated in the Results and illustrated in Figure S3, the threshold T_{assign} for an optimal trade-off between AR and ER will depend amongst others on the proportions of different categories of relatives in the sample: in Pedigree I, lower values result in somewhat lower ER and considerably better AR, while in Pedigree III higher values of T_{assign} result in lower ER and hardly affect AR (Figure S3a). As the proportion of different relatives are typically not known *a priori*, one approach would be to run Sequoia with different values of T_{assign} and/or T_{filter}, and use the run with the highest total likelihood. Encouragingly, when

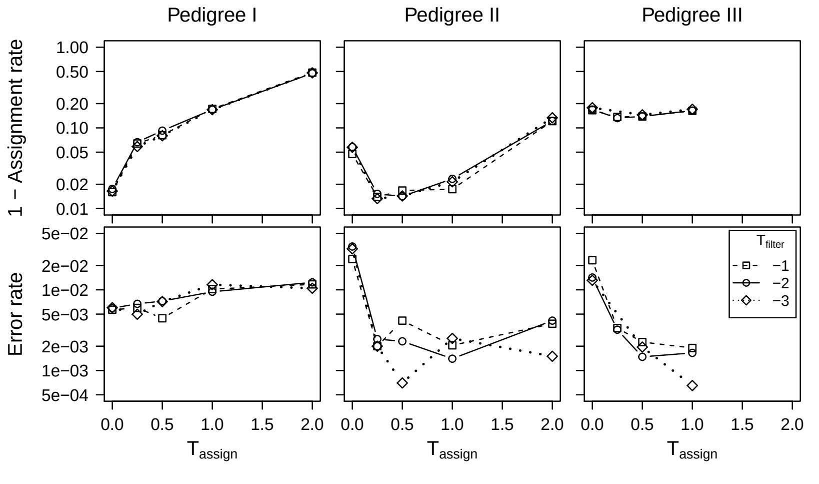

(a) Effect of T_{assign} (x-axis) and T_{filter} (lines, symbols) on AR and ER in Pedigree I–III. Squares, dashed lines: $T_{filter} = -1$; circles, solid lines: $T_{filter} = -2$ (default used in Results); diamonds, dotted lines: $T_{filter} = -3$. Simulated datasets included 200 SNPs and 0% (Ped I) or 60% (Ped II–III) of parents genotyped, points show averages over 5 replicates.

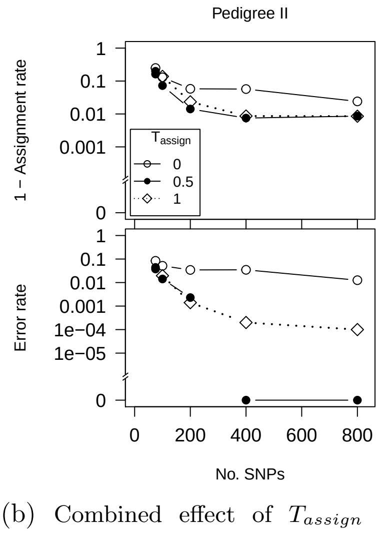

(b) Combined effect of T_{assign} (lines) and SNP number (x-axis) on AR and ER in Pedigree II. Squares, dashed lines: $T_{assign} = 0$; circles, solid lines: $T_{assign} = 0.5$ (default used in Results); diamonds, dotted lines: $T_{assign} = 1$. 60% of parents were assumed genotyped, and points show averages over 5 replicates.

Figure S3: Effects of T_{filter} and T_{assign} on AR and ER.

Table S3: Alternative algorithm orders considered. 'Rounds' refers to the iterations in which each step is executed.

	Default		A1		A2		A3		A4	
	Order	Rounds	Order	Rounds	Order	Rounds	Order	Rounds	Order	Rounds
Sibling pairs	0		0		0		0		0	
Cluster siblings	1		1		1		1		1	
Merge sibships	2	all	2	all	2	all	2	all	6	all
Add to sibships	3	>1	3	all	3	>1	3	>1	7	>1
Sibship parents	4	>1	4	all	4	>1	6	>1	2	>1
Parentage	5	>1	5	all	5	>1	7	>1	3	>1
GG pairs	6	>2	6	>1	6	>1	4	>2	4	>2
Sibship GG	7	>1	7	all	7	all	5	>1	5	>1

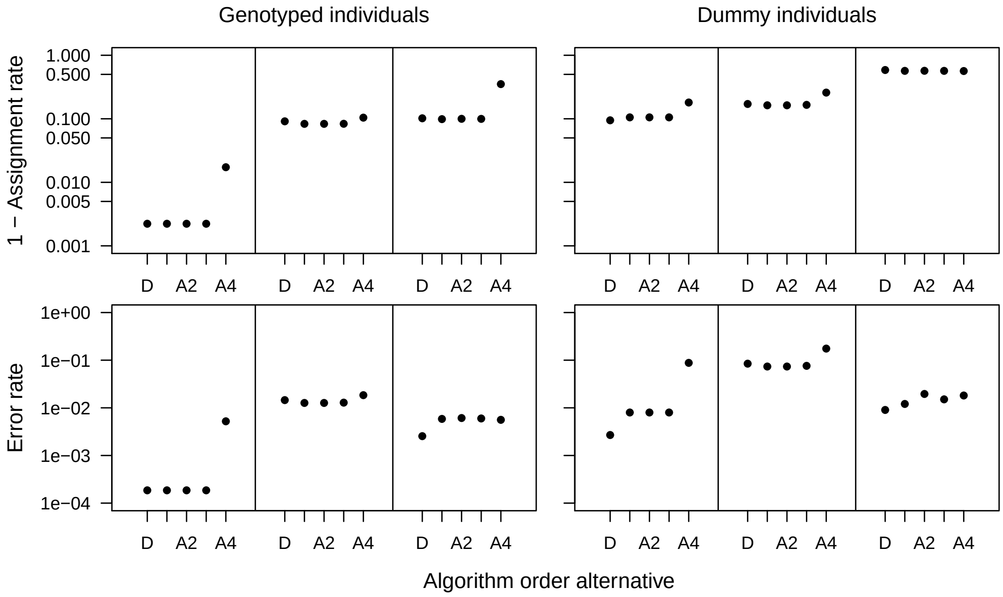

Figure S4: Effects of changing the algorithm order (x-axes, as in Table S3, D=Default) on AR and ER, when run on identical datasets based on Pedigree II and 200 SNPs (left-most in each panel), Pedigree II and 100 SNPs (middle) or Pedigree III and 200 SNPs. Averages over 3 replicate datasets shown.

analysing the same simulated data using different parameter values, the run with the highest total likelihood seems to consistently have the lowest error rate, although this was explored in detail only for assuming a monogamous breeding system or not (Figure S9 below). For Pedigree II, $T_{assign} = 0.5$ resulted in lower or approximately equal ER and similar or better AR than $T_{assign} = 0$ or $T_{assign} = 1$, irrespective of marker number (Figure S3b).

S2.2 Algorithm order

As there typically are only a handful of iterations until convergence, and each step assumes that all earlier made assignments are correct (except durring parentage assignment), the order of steps within each iteration is likely to be relevant. The order was chosen to reduce error rates to a minimum, especially in the first iteration, as any error may potentially cause a snowball effect of subsequent errors (although this did not occur with any of the tested datasets in the current version of the algorithm, due to numerous build-in fail-safes). As explained in the main text, several rounds of parentage assignment are therefore performed prior to any sibship clustering. The default order after parentage assignment is shown in the first columns of Table S3, as well as a few alternatives. For example, in alternative A3 assignment of grandparents assignment was performed prior to assignment of parents to sibships and singletons.

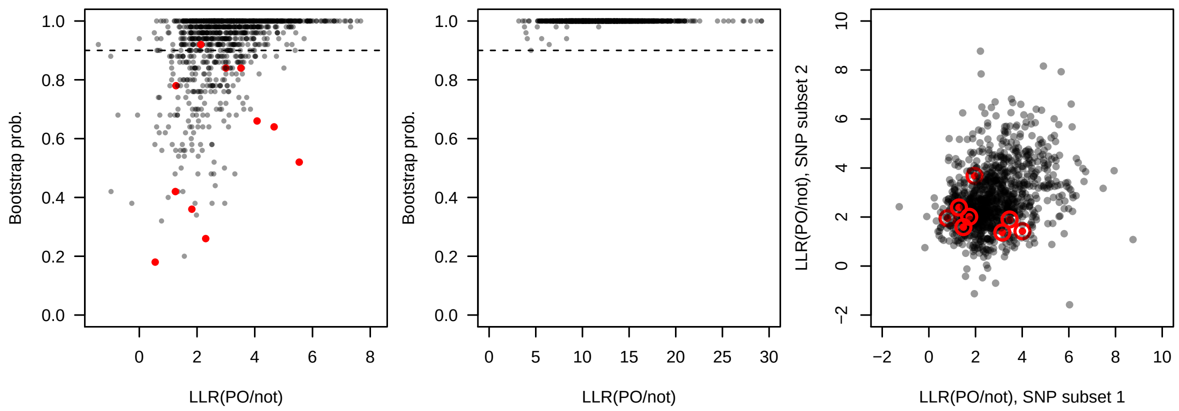

Figure S5: Relationship between $\Lambda_{PO/\vee}$ and the proportion of bootstrap replicates in which that parent was assigned for Pedigree II with 60% of parents genotyped, using (a) 100 SNPs or (b) 400 SNPs and 50 bootstrap replicates. Red points indicate erroneous assignments. In panel (c), the LLR(parent/not parent) are shown of pedigrees inferred using two subsets of the SNPs of a single dataset, with red points again indicating erroneous assignments, which all resulted in mismatches between the two inferred pedigrees (red circles), i.e. errors differed between the two data subsets.

The default order always resulted in an equal or lower ER and an equal or higher AR than all of the alternatives (Figure S4). There is no difference between alternatives A1–A3 for the comparatively simple Pedigree II (left and middle sections of each panel), while there is a difference for the complex Pedigree III (right sections). There, changing the algorithm order especially increases the number of wrongly assigned parents of dummy individuals (i.e., grandparents of sibships).

S2.3 Assignment confidence

To quantify the probability that inferred pedigree links are correct, one can use a 'forward' approach, starting from presumed pedigrees, or a 'backward' approach, starting from the genetic and phenotypic data (Morrissey *et al.*, 2007).

Bootstrap is an example of the latter, as is common in the inference of phylogenies since proposed by Felsenstein (1985). Then, in each of typically 20–200 bootstrap iterations, the genotype data is permuted by sampling L loci *with replacement* from the L available loci, and the pedigree is re-reconstructed based on the permuted genotypes. However, during initial explorations 25% of correctly assigned parents had a bootstrap probability below 90% when using 100 SNPs (Figure S5a). This overly conservative estimate is probably related to the increased dependency between markers in each bootstrap replicate, as some markers are duplicated. Instead, one could split the markers into two equal sized subsets, and run sequoia on each half. When trialling this on $L1 = L2 = 100$ SNPs, none of the incorrect assignments based on either SNP subset was repeated using the other subset. However, consensus assignment rate over the two subsets will be lower than when all SNPs are used, and the error rate did not appear to differ. Splitting the data repeatedly and using a larger subset of markers (e.g. 80%) for pedigree inference seems a promising albeit time-consuming avenue to estimate assignment confidence, but requires more detailed investigation.

An example of a forward approach simulation is implemented in Cervus, where founder genotypes are generated according to population allele frequencies, and offspring genotypes derived by Mendelian sampling (Marshall *et al.*, 1998). A similar approach is used here to estimate assignment and error rates (described below, 'Simulation of SNP data')

Alternatively, one can use smart algorithms to estimate the probability that at least one candidate parent of an individual, which is not an actual parent, has a value $\Lambda_{PO/\vee}$ that exceeds the value of the assigned parent, as for example in Anderson (2012).

References

Anderson EC (2012) Large-scale parentage inference with SNPs: an efficient algorithm for statistical confidence of parent pair allocations. *Statistical applications in genetics and molecular biology*, **11**, 12.

Anderson EC, Garza JC (2006) The power of single-nucleotide polymorphisms for large-scale parentage inference. *Genetics*, **172**, 2567–2582.

Epstein MP, Duren WL, Boehnke M (2000) Improved inference of relationship for pairs of individuals. *The American Journal of Human Genetics*, **67**, 1219–1231.

Felsenstein J (1985) Confidence limits on phylogenies: an approach using the bootstrap. *Evolution*, S. 783–791.

Hadfield JD, Richardson DS, Burke T (2006) Towards unbiased parentage assignment: combining genetic, behavioural and spatial data in a bayesian framework. *Molecular Ecology*, **15**, 3715–3730.

Hoffman JI, Tucker R, Bridgett SJ, Clark MS, Forcada J, Slate J (2012) Rates of assay success and genotyping error when single nucleotide polymorphism genotyping in non-model organisms: a case study in the antarctic fur seal. *Molecular ecology resources*, **12**, 861–872.

Marshall TC, Slate JBKE, Kruuk LEB, Pemberton JM (1998) Statistical confidence for likelihood-based paternity inference in natural populations. *Molecular ecology*, **7**, 639–655.

Morrissey MB, Wilson AJ, Pemberton JM, Ferguson MM (2007) A framework for power and sensitivity analyses for quantitative genetic studies of natural populations, and case studies in Soay sheep (*ovis aries*). *Journal of evolutionary biology*, **20**, 2309–2321.

Stopher KV, Nussey DH, Clutton-Brock TH, Guinness F, Morris A, Pemberton JM (2012) Remating across years and intralineage polygyny are associated with greater than expected levels of inbreeding in wild red deer. *Journal of Evolutionary Biology*.

Wang J (2004) Sibship reconstruction from genetic data with typing errors. *Genetics*, **166**, 1963–1979.

S2.4 Additional tables and figures

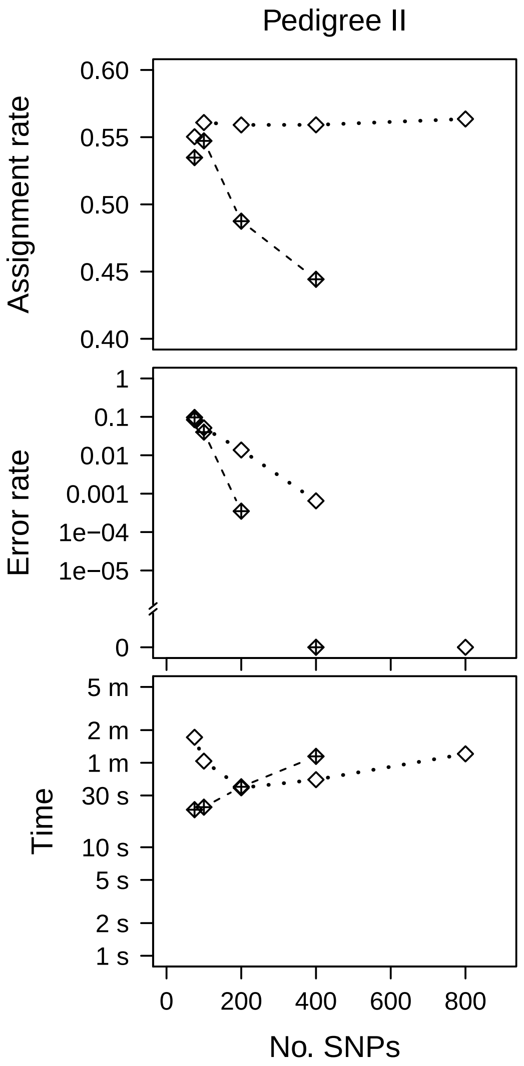

Figure S6: Difference in performance of parentage assignment by FRANz when using the default settings (open diamonds) or using the option assisted by full sib clustering ('–fullsibtest' and '–fullsibparental'; crossed diamonds). Performance of FRANz in Pedigree I was near perfect, leaving no space for possible improvement by sibship clustering, while pedigree III contains very few full siblings.

Table S4: Datapoints underlying Figure 6: Effect of the number of independent SNPs on AR, ER and computational times in parent assignment using Sequoia, opposite-homozygosity based exclusion (OH-Excl) or FRANz. All parental genotypes are assumed known in simulated datasets based on three different pedigree structures. Each value denotes the average over 20 independent simulations.

Pedigree		Assignment rate			Error rate			Computational time*		
	SNPs	FRANz	Sequoia	OH-Excl	FRANz	Sequoia	OH-Excl	FRANz	Sequoia	OH-Excl
I	75	1	0.994	0.081	0	4.8E-04	0.92	03:01.0	00:13.3	00:01.4
	100	1	0.998	0.425	0	4.3E-05	0.57	01:25.2	00:07.1	00:01.4
	200	1	1	1	0	0	1.9E-04	00:32.3	00:09.7	00:01.4
	400	1	1	1	0	0	0	00:49.4	00:15.9	00:01.6
	600	1	1	1	0	0	0	01:12.0	00:22.4	00:01.7
	800	1	1	1	0	0	0	01:25.8	00:29.0	00:01.8
II	75	1	0.995	0.125	1.0E-04	1.6E-03	0.84	03:17.1	00:12.2	00:01.1
	100	1	0.999	0.262	0	2.5E-04	0.71	01:46.0	00:07.0	00:01.1
	200	1	1	0.936	0	0	6.2E-02	00:33.8	00:07.0	00:01.2
	400	1	1	0.999	0	0	7.5E-04	00:42.5	00:11.1	00:01.3
	600	1	1	1	0	0	0	01:06.2	00:15.6	00:01.3
	800	1	1	1	0	0	0	01:17.0	00:20.1	00:01.4
III	75	0.997	0.978	0.129	2.6E-02	5.7E-03	0.63	08:03.0	00:49.5	00:01.5
	100	1	0.983	0.424	7.8E-03	6.0E-04	0.42	03:20.1	00:15.0	00:01.6
	200	1	0.983	0.974	4.0E-03	0	7.0E-03	01:03.7	00:14.3	00:01.6
	400	1	0.983	0.983	3.8E-03	0	0	00:58.3	00:22.2	00:01.8
	600	1	0.983	0.983	3.8E-03	0	0	01:20.9	00:31.5	00:01.8
	800	1	0.983	0.983	3.8E-03	0	0	01:43.7	00:41.5	00:02.1

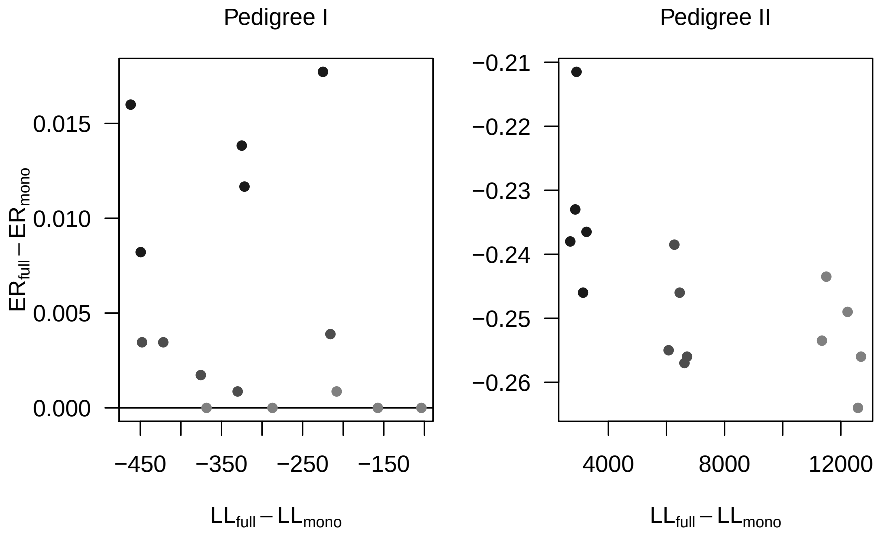

Figure S9: Difference in error rate (y-axis) and total log likelihood (x-axis) when running Sequoia on ‘full’ or when assuming monogamy (‘mono’), on the same dataset. The reconstructed pedigree with the highest likelihood always has fewer than, or an equal number of errors as, the alternative; i.e. erroneous assignments due to enforcing monogamy are not spuriously increasing the likelihood.

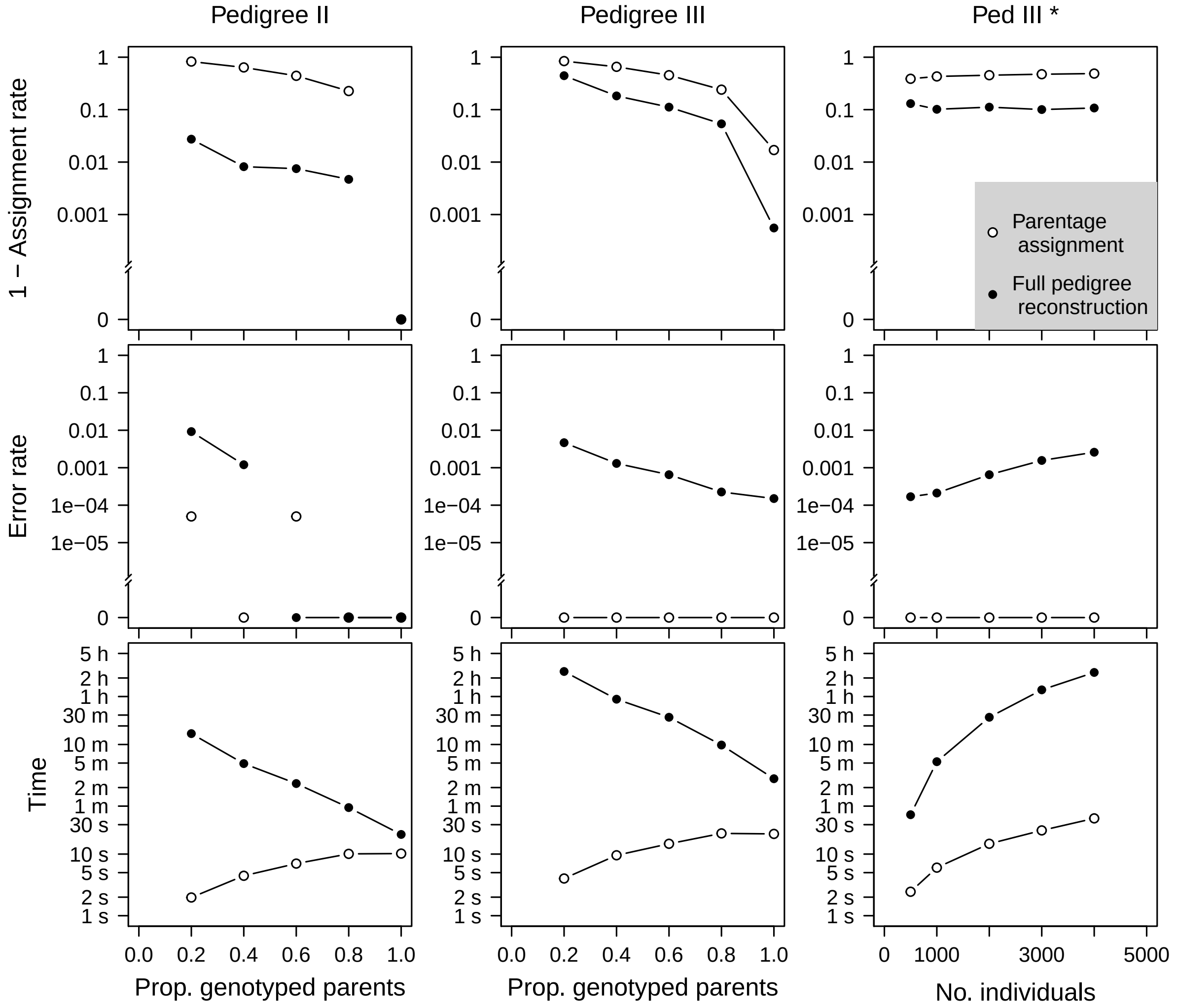

Figure S10: Effect of the proportion of genotyped parents in Pedigrees II and III, as well as the effect of pedigree size (more individuals confounded with a deeper pedigree; 60% of parents genotyped) on AR, ER and computational time; averages over 10 replicates are shown. Filled circles: Sequoia, open circles = Sequoia's parentage assignment only. Computational time increased with the number of individuals in an approximately quadratic fashion, as one of the most computationally intensive steps is the identification of sibling pairs among the approximately $N^2/2$ pairs of individuals. Another strong determinant of the computational time is the number of sibships S, as the merging step will consider around $(S/2)^2$ combinations, and the subsequent step adding individuals to existing sibships will consider order SN combinations.

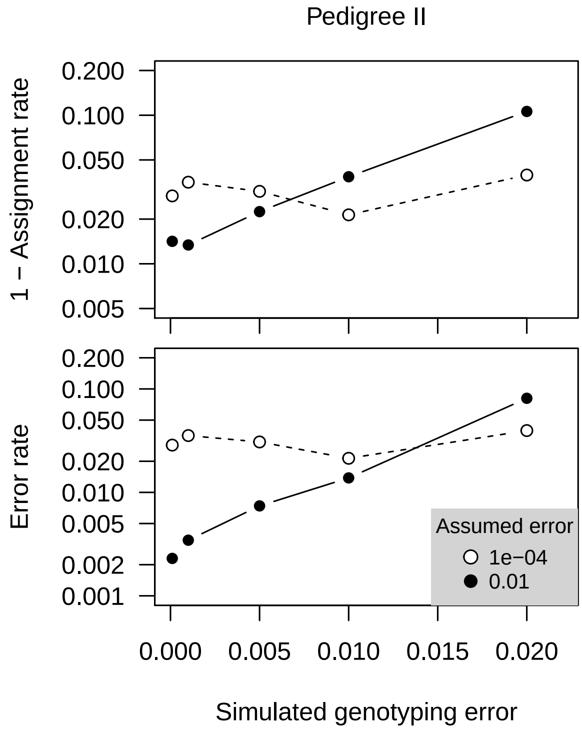

Figure S7: Effect of simulated genotyping error rate (x-axis) and assumed genotyping error rate (lines and symbols, filled circles: 1e-4 (default), open circles: 1e-2) on AR and ER in Pedigree II with 200 SNPs and assuming 60% of parents genotyped.

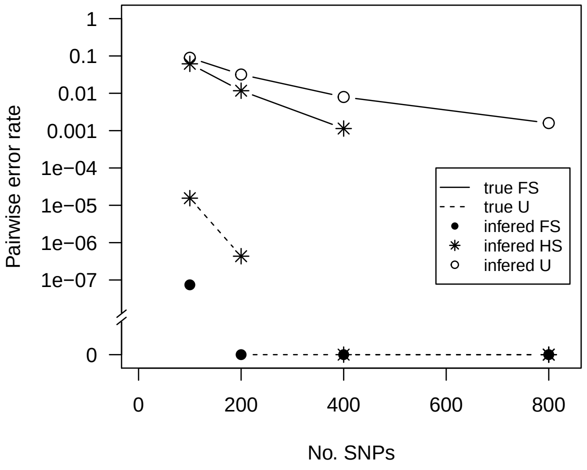

Figure S8: Proportion of misclassified pairs in Pedigree I among FS (solid lines) and U (dashed) pairs. Filled points denote classification as FS, asterisks as HS, and open points as U; averages over 10 replicate runs are shown. Note that pedigree I consists of 1983 FS pairs and 6.6×10^5 U pairs, such that erroneous assignment of one individual to a full sib family with, say, 2 members results in an pairwise error rate $P(\text{FS}|\text{U}) = 3.03 \times 10^{-6}$.
