# Supplementary material for: Pedigree reconstruction from SNP data: parentage assignment, sibship clustering and beyond
Source: Mol Ecol Resour. 2017 Apr 6;17(5):1009–24. doi: 10.1111/1755-0998.12665 (PMC6849609; doi:10.1111/1755-0998.12665)
Supplement: Supplementary file 2 — Appendix S2 Sequoia R vignette. [file MEN-17-1009-s002.pdf]

# sequoia

Jisca Huisman
jisca.huisman@gmail.com

February 6, 2017

`Sequoia` provides a method to reconstruct multi-generational pedigrees based on SNP data, as described in [1]. The bulk of the algorithm is written in Fortran, to minimise computation times. An example R script is shown, followed by detailed description of each step.

## Background

Data on hundreds of SNPs provides detailed information on the relationships between individuals, but with such large amounts of data pedigree reconstruction using MCMC or other approaches with many thousands of iterations are extremely time consuming. Instead, `Sequoia` is a conservative hill-climbing algorithm, asymptoting to a high-likelihood pedigree in a handful of iterations. Explicit consideration of the likelihoods of alternative configurations between pairs or groups of individuals before making an assignment reduces the number of false positives compared to methods relying on the likelihood ratio parent-offspring/unrelated (LLR(PO/U)) only [4].

# Contents

**1 Input** **2**
- 1.1 Life history data . . . 2
- 1.2 Genotype data . . . 3

**2 Running Sequoia** **4**
- 2.0 Input parameters . . . 5
- 2.1 Check for duplicates . . . 7
- 2.2 Age difference based prior . . . 7
- 2.3 Parentage assignment . . . 9
- 2.4 Sibship clustering & the rest . . . 10

**3 Output check** **12**
- 3.1 Comparison with previous pedigree . . . 12
- 3.2 Estimating confidence probabilities . . . 15
- 3.3 Comparison pedigree-based and genomic relatedness . . . 16

## Example

An example pedigree and associated life history data are provided with the package, which can be used to try out the steps detailed in this vignette. This pedigree consists of 5 generations with interconnected half-sib clusters (Pedigree II in [1]).

```
> install.packages("sequoia")  # only required first time
> setwd("E:/Sequoia/test")     # set the working directory
> library(sequoia)             # load the package
> #
> # get the example pedigree and life history data
> data(Ped_HSg5, LH_HSg5)
> tail(Ped_HSg5)
> #
> # simulate genotype data for 200 SNPs
> Geno <- SimGeno(Ped = Ped_HSg5, nSnp = 200)
> #
> # run sequoia - duplicate check & parentage assignment only
> ParOUT <- sequoia(GenoM = Geno,
+                   LifeHistData = LH_HSg5,
+                   MaxSibIter = 0)
> #
> # run sequoia - sibship clustering & grandparent assignment
> SeqOUT <- sequoia(GenoM = Geno,
+                   SeqList = ParOUT,
+                   MaxSibIter = 5)
> #
> # compare the assigned real and dummy parents to the true pedigree
> PedCompare(Ped1 = Ped_HSg5, Ped2 = SeqOUT$Pedigree)
> #
> # save results
> save(SeqOUT, file="Sequoia_output_date.RData")
> write.table(SeqOUT$Pedigree, file="Pedigree_date.txt",
+             sep="\t", row.names=FALSE, quote=FALSE)
>
```

# 1 Input

## 1.1 Life history data

The life history data (`LifeHistData`) should be provided as a dataframe with three columns:

- ID: up to 30 characters long, no spaces

- Sex: 1 = female, 2 = male, other numbers = unknown
- Year of birth (or hatching). In species with more than one generation per year, a finer time scale than year of birth ought to be used, ensuring that parents are never born within the same time unit as their putative offspring.

Ideally this basic life history information is provided for all genotyped individuals, but this is not strictly necessary. This dataframe may include many more individuals than the genotype data, or in a different order.

## 1.2 Genotype data

The SNP data should be coded with one line per individual, and one column per SNP, with each SNP is coded as 0, 1, 2 copies of the reference allele, or missing (-9). This matrix can be provided as `GenoM`, with individual IDs as the rownames. Alternatively a text file `GenoFile` can be specified with one column for IDs followed by one column per SNP, and no header row. Then, the genotype data is never loaded into R, as this can take up a lot of memory for very large datasets. The necessary file format can for example be obtained using PLINK [3] in combination with `sequoia`'s `GenoConvert`, as described below. `GenoConvert` can also convert Colony input files.

### 1.2.1 Real data - Selection of SNP markers

Using tens of thousands of SNP markers for pedigree reconstruction is unnecessary, will slow down computation, and may even hamper inferences by their non-independence. Rather, a subset of SNPs in low linkage disequilibrium (LD) with each other, and with high minor allele frequencies (MAF $> 0.3$), ought to be selected first if more than a few hundred SNPs are available. The calculations assume independence of markers, and While low (background) levels of LD are unlikely to interfere with pedigree reconstruction, high levels may give spurious results. Markers with a high MAF provide the most information, as although rare allele provide strong evidence when they are inherited, this does not balance out the rarity of such events.

Creating a subset of SNPs can be done conveniently using PLINK (`https://www.cog-genomics.org/plink2`), using for example the command

```
plink --file mydata --maf 0.4 --indep 50 5 2
```

which will create a list of SNPs with a minor allele frequency of at least 0.4, and which in a window of 50 SNPs, sliding by 5 SNPs per step, have a VIF of maximum 2. VIF, or variance inflation factor, is $1/(1-r^2)$. It is advised to 'tweak' the parameter values until a set with a few hundred SNPs (300-700) is created. For further details, see `https://www.cog-genomics.org/plink2/ld#indep`.

The resulting list ('plink.prune.in') can be used to create the genotype file used as input for Sequoia, with SNPs codes as 0, 1, 2, or NA, with the command

```
plink --file mydata --extract plink.prune.in --recodeA --out
 inputfile_for_sequoia
```

in command prompt, which is on a windows machine equivalent to

```
> system("cmd", input = "plink --file mydata --extract plink.prune.in
+   --recodeA --out inputfile_for_sequoia")
```

in R. This will create a file with the extension .RAW, which can be converted to the required input format using

```
> GenoM <- GenoConvert(InFile = "inputfile_for_sequoia.raw",
+                      OutFile = NA)
```

This function can also convert from files in two-columns-per-SNP format, as used by e.g. Colony.

### 1.2.2 Simulating SNP data

When SNP data is not (yet) available, but an approximate pedigree is, it is possible to test `sequoia` on a simulated dataset. This may be useful to for example explore the number of markers required to reliably infer a pedigree of that particular structure. Alternatively, this can be used to estimated the pedigree-wide error rate of an inferred pedigree.

The function `SimGeno()` lets the user specify the average proportion of missing genotypes per individual (`MisHQ`), the genotyping error rate (`ErHQ`), and the fraction of known parents (in the supposed 'true' pedigree) which have not been genotyped (`ParMis`). Moreover, the data can be made to contain a fraction of low-quality samples (`PropLQ`, with associated `MisLQ` and `ErLQ`), to assess whether inclusion of samples which did not pass stringent quality control would improve or hamper pedigree reconstruction.

# 2 Running Sequoia

Under the hood, `sequoia` consists of four sub-programs:

1. **Duplicates:** Check for duplicate entries in the genotype and life history data
2. **Agepriors:** Initiation or calculation of age-difference based priors
3. **Parentage:** Parentage assignment (assign genotyped parents to genotyped focal individuals)

4. **Sibships:** Clustering of half- and full-siblings, grandparent-assignment to singletons and sibships, and identification of avuncular relationships between sibships (jointly referred to as 'Sibships' for brevity)

which all return their output to a single list, with the elements as listed in Table 2.

## 2.0 Input parameters

The parameter values that can be changed are as follows:

**DummyPrefix** The prefixes for dummy individuals (parents assigned to sibship clusters) can be altered to avoid confusion with IDs of real individuals.

**Err** The genotyping error rate assumed, typically probably around 1E-4 to 1E-3. The error model is given in Table 1; other error structures could easily be implemented but are currently not user-settable.

Table 1: Default probabilities used of observing genotype $X$, conditional on actual genotype $x$.

| | $X$ | | |
|---|---|---|---|
| $x$ | 0 | 1 | 2 |
| 0 | 1-$\epsilon$ | $\epsilon$ | 0 |
| 1 | $\epsilon/2$ | 1-$\epsilon$ | $\epsilon/2$ |
| 2 | 0 | $\epsilon$ | 1-$\epsilon$ |

**GenoFile** The name of the genotype file. When not in the current working directory (`getwd()`), provide the full path (with forward slashes).

**GenoM** The genotype matrix. Can be read in from a genotype file in the prescribed format using

```
> GenoM <- as.matrix(read.table("SimGeno.txt", row.names=1))
```

**LifeHistData** The dataframe with IDs, Sex and Birth or hatching year, in that order. The column names are ignored. It is possible to read this in directly from a text file:

```
> ParOUT <- sequoia(GenoM = GenoM,
+                   LifeHistData = read.table("LifeHistFile.txt",
+                                             header=TRUE),
+                   MaxSibIter=0)
```

**MaxMismatch** The maximum number of loci at which candidate parent and offspring are allowed to be opposite homozygotes, used to filter out highly unlikely pairs. Note that the actual upper limit used is MaxOH = MaxMismatch + ceiling(Err * nSnp).

**MaxSibIter** The number of iterations of sibship clustering. As this is by far the most time consuming step, and may take several hours for large datasets, it would be wise to first run with `MaxSibIter=0` so that only the much faster parentage assignment is performed, and inspect the output. If the total likelihood asymptotes before `MaxSibIter` is reached, the algorithm is terminated and the results returned.

**MaxSibshipSize** Maximum number of offspring for a single individual. A generous safety margin is advised of at least twice the biologically plausible maximum.

**Tassign** Threshold log10-likelihood ratio (LLR) required for acceptance of proposed relationship, relative to next most likely relationship. Must be zero or positive, with higher values resulting in more conservative assignments.

**Tfilter** Threshold LLR between a proposed relationship versus unrelated, to select candidate relatives. For the filtering step, the LLR is calculated without conditioning on currently assigned parents ($\Lambda^*_{R/U}$ in [1]), to speed up computation. `Tfilter` is typically a negative value, and more negative values may decrease non-assignment (preventing filtering out of true relatives), but will increase computational time.

**Complexity** When it is known that the dataset contains only monogamous matings, the assignment rate can be improved by using the option `Complexity="mono"`. <under development . . . >

### 2.0.1 Re-use of previous output

The parameter values used as arguments when calling `sequoia` will be returned in the list element `Specs`. These settings can be re-used in a subsequent run using

```
> load("Sequoia_output_date.RData")  # if it was saved to disk
> SeqOUT2 <- sequoia(SeqList = list(Specs = SeqOUT1$Specs))
```

which will then not use any of the other parameter values provided, *except* `MaxSibIter`. One may change the values in `Specs`, ensuring to not alter the names of the components.

It is also possible to re-use the entire output list,

```
> SeqOUT2 <- sequoia(SeqList = SeqOUT1)
```

Table 2: Output from Sequoia, returned within a named list.

| Output | Description |
|---|---|
| AgePriors | Age-difference based prior probabilities |
| DummyIDs | Details per half-sib cluster |
| DupGenoID | Duplicated IDs in genotype data |
| DupGenotype | (near) Duplicated genotypes |
| DupLifeHistID | Duplicated IDs in life history data |
| LifeHist | sex and birth year data |
| MaybeParent | Non-assigned likely PO pairs |
| MaybeRel | Non-assigned likely relatives |
| NoLH | IDs in genotype data not present in life history data |
| Pedigree | Pedigree |
| PedigreePar | Scaffold pedigree |
| Specs | Parameter values |
| TotLikParents | Total likelihood during parentage |
| TotLikSib | Total likelihood during sib clustering |

which will use `AgePriors` in SeqOUT1, as well as `PedigreePar` (and thus parentage assignment will not be run).

## 2.1 Check for duplicates

The data may contain positive controls, as well as other intentional and unintentional duplicated samples. Sequoia quickly searches the data for identical genotypes and IDs, which ought to be removed prior to parentage assignment. It allows a few mismatches between the genotypes (`MaxMismatch`), with or without the same individual ID. Note that when the number of SNPs is limited, very inbred individuals may be nearly indistinguishable from their parent(s); such individuals should not be excluded.

This function additionally searches the life history data for duplicated entries, and will also return a vector of individuals included in the genotype data, but not in the life history data (`NoLH`). The latter is merely a service to the user; individuals without life history information can often be successfully included in the pedigree.

## 2.2 Age difference based prior

The list element `AgePriors` contains 8 columns, and as many rows as the birth year range detected in the life history data. It initially only indicates whether a given relationship is biologically possible (1) or not (0) for a given age difference between individuals. The first row is for individuals born in the same year, the second row for individuals born one year apart, etc. The columns are labelled for various relationship categories, with M = mother, P = father, MS = maternal sibling, PS = paternal sibling, MGM = maternal grandmother, PGF = paternal grandfather, MGF = maternal grandfather and paternal grandmother, and AU = avuncular (niece/nephew –

aunt/uncle).

For example, the first value in the column 'MS' can be interpreted as 'if I were to pick two individuals born in the same year, and two individuals from my sample at random, how much more likely are the first pair to be maternal siblings, compared to the second pair?' Values below 1 indicate less likely, and values above 1 more likely. For MS, PS and AU absolute age differences are used (with overlapping generations, nephews may be older than their aunts), while parents and grandparents are necessarily older than their (grand-)offspring (categories M, P, MGM, PGF and MGF).

These age-difference based priors are by default automatically updated after parent-age assignment, based on the empirical distribution of age differences between individuals and their assigned fathers and mothers. This update is prevented when `SeqList` is provided and contains an element 'AgePriors'.

Table 3: Behaviour when 'AgePriors' and/or 'PedigreePar' are provided in 'SeqList'. –: not provided / not run, AP1 = simple age prior, AP2 = parents-based age prior

| in SeqList | | Use ... | |
|---|---|---|---|
| AgePriors | PedigreePar | Parentage | Sibships |
| – | – | AP1 | AP2 |
| AP* | – | AP* | AP2 |
| – | Y | – | AP2 |
| AP* | Y | – | AP* |

As with the parameter specifications, feel free to alter the values to match the biological characteristics of the species. The number of rows may be increased (but not decreased below the age range amongst the genotyped individuals according to the provided life history data), in which case `Specs["nAgeClasses"]` should be updated to match the new number of rows. For example, for a species with strictly non-overlapping generations, one may wish to alter the AgePriors to

| MS | PS | MGM | PGF | MGF | UA | M | P |
|---|---|---|---|---|---|---|---|
| 1 | 1 | 0 | 0 | 0 | 0 | 0 | 0 |
| 0 | 0 | 0 | 0 | 0 | 1 | 1 | 1 |
| 0 | 0 | 1 | 1 | 1 | 0 | 0 | 0 |
| 0 | 0 | 0 | 0 | 0 | 0 | 0 | 0 |
| 0 | 0 | 0 | 0 | 0 | 0 | 0 | 0 |

and express the 'birth years' in the life history data as generation numbers. This can be done as follows

```
> AP <- as.matrix(SeqOUT$AgePriors)
> AP[AP>0] <- 0
> AP[1,c("MS", "PS")] <- 1
> AP[2,c("M", "P", "UA")] <- 1
> AP[3,c("MGM", "PGF", "MGF")] <- 1
> SeqOUT2 <- sequoia(SeqList=list(Specs=SeqOUT$Specs, AgePriors=AP),
+                    MaxSibIter = 0)
```

Note that any identified parent-offspring pairs which are not a single generation apart will be flagged (see ‘Non-assigned parent-offspring pairs’ below).

## 2.3 Parentage assignment

Parentage assignment is quick, and for example takes less than a minute for an empirical dataset with 2,500 genotyped individuals on a laptop with an intel i7 2.3 GHz CPU and 8GB RAM.

### 2.3.1 PedigreePar

This scaffold pedigree contains columns with

- IDs of the individual, its assigned dam (mother) and sire (father);
- The log10 likelihood ratio (LLR) of the dam, sire and the parent pair; this is the ratio between the likelihood of the assigned parent being the parent, versus the most likely alternative type of relative to the focal individual. The alternative relationships considered are full sibling (FS), half sibling (HS), grandparent (GG), full avuncular (FA), half avuncular / great-grandparental / cousin (HA), or unrelated (U).
- The number of loci at which the offspring and the assigned dam or sire are opposite homozygotes;
- The row number in the genotype matrix of the offspring, dam and sire; used for subsequent sibship clustering.

The parental LLRs are calculated after the likelihood has reached an asymptote, and are conditional on all other links in the reconstructed pedigree. They are calculated by removing the parent(s), and re-calculating the likelihoods of the various relationships between the focal individual and the parent. The pair LLR is relative to the most likely assignment of a single parent (or no parent). Note that the reported LLR differs from for example Cervus [2], which instead returns the natural log of the ratio between the probability that the assigned parent is the parent, versus that the next most likely candidate is the parent.

Some parents may have a very small or even negative single-parent LLR, but the LLR of the parent pair should ideally always be positive. For full sibling pairs and dummy-parents of dummy-individuals this is not always the case, due to some approximations used when calculating the parental LLR (which are not used during the assignment steps). It is however probably worthwhile to compare assignments with low or negative LLRs with a previous pedigree or the genomic relatedness, as described at the end of this document.

If some of the LLRs are very large negative or positive numbers, please send a bug report to `jisca.huisman@gmail.com` with a short description of your dataset.

#### 2.3.2 MaybeParent

Sometimes probable or definite parent-offspring pairs are identified which could not be assigned as such in the pedigree. This includes both cases where the pair is more likely to be parent-offspring than unrelated (LLR_Rx_U > Tfilter), but where it cannot be excluded that they are otherwise related (LLR_R1_R2 < Tassign), indicated by FS (full siblings), FA (full avuncular), XX (unclear) etc. in the column "TopRel" (most likely relationship). '2nd' indicates that the pair are most likely to be second degree relatives, but that it is impossible to distinguish between HS, GG and FA (which would require at least 1 parent assigned to each individual, see [1]). For these cases, LLR_R1_R2 gives the LLR between being 2nd degree relatives versus the most likely of PO, FS, HA or U.

If PO (parent-offspring) had the highest likelihood, they were non-assigned either because it was not possible to tell which of the two was the parent and which was the offspring, due to either or both individuals having an unknown birth year (coded as AgeDif = 999), or because it was not possible to tell whether the candidate parent was the mother or the father (Sex2 = 3). These situations can be remedied by providing estimated birth years or guessed genders in the life history data for the individuals involved, and re-running `sequoia`.

#### 2.3.3 TotLikParents

A vector with the initial total likelihood of the data, assuming a random draw from a random mating population at Hardy-Weinberg equilibrium, followed by the total likelihood at the end of each iteration.

### 2.4 Sibship clustering & the rest

Sibship clustering, amongst those individuals which have not been assigned two genotyped parents, is performed when `MaxSibIter`> 0. Sibship clustering may take from a few seconds to a few hours, depending on the number of individuals without a parent, the number of sibships that is being clustered, and their degree of interconnection. Calculation of the parental likelihoods and the check for non-assigned potential relatives (during 'Creating output ...') may take considerable time too.

The total likelihood typically asymptotes within five to ten iterations, even for complex pedigrees. When an asymptote is reached before `MaxSibIter`, a final iteration with stronger dependence on the age prior is ran, (dummy)parental likelihoods are calculated, and the algorithm is terminated. Whether or not the asymptote has been (nearly) reached can be seen from `TotLikSib`.

### 2.4.1 Pedigree

The main output of the sibship clustering is returned in 'Pedigree', which is highly similar to 'PedigreePar' (see earlier). It contains all assigned real parents, as well as the dummy parents assigned during sibship clustering. Note that dummy individuals are also assigned as the 'in-between' individual of identified grandparent — grand-offspring pairs. Dummy individuals are appended at the bottom of this pedigree with their assigned parents, i.e. the sibship's assigned grandparents.

### 2.4.2 DummyIDs

To each cluster of half-siblings a "dummy" parent is assigned, denoted by increasing numbers, by default with prefix 'F' for females and 'M' for males. In 'DummyIDs', for each dummy individual its assigned parents are listed (as in the Pedigree), as well as its sex, its estimated birth year (based on the birth years of its offspring and parents; point estimate, upper and lower bound of 95% confidence interval are provided), the number of assigned offspring, and the IDs of its assigned offspring.

### 2.4.3 MaybeRel

This dataframe contains for example half-siblings where it could not be determined whether they are maternal or paternal half-siblings (currently no half-sibships of unknown maternal/paternal type are created). 'LLR_Rx_U' gives the maximum of LLR(PO/U), LLR(FS/U), LLR(HS/U); note that LLR(HS/U) = LLR(GG/U) = LLR(FA/U), as these are calculated not conditional on any assigned parents (see [1] for details). 'Relx' denotes whether this maximum was for PO, FS, or HS.

### 2.4.4 TotLikRel

As 'TotLikParents', for sibship clustering. One can do a visual check if the asymptote has been (nearly) reached within 'MaxSibIter' using

```
> TLL <- c(SeqOUT$TotLikParents, NA, SeqOUT$TotLikSib)
> xv <- c(paste0("p", 1:length(SeqOUT$TotLikParents)-1), " ",
+         paste0("s", 1:length(SeqOUT$TotLikSib)-1))
> plot(TLL, type="b", xaxt="n", xlab="Round")
> axis(1, at=1:length(TLL), labels=xv)
```

# 3 Output check

## 3.1 Comparison with previous pedigree

Often times, a (part) pedigree is already available to which one wants to compare the results, for example consisting of maternal links, deduced from observations in the field. The function `PedCompare()` performs such comparisons, and takes as arguments the 'true' pedigree and the inferred pedigree:

```
> compareOUT <- PedCompare(Ped1 = Ped_HSg5, Ped2 = SeqOUT$Pedigree)
```

The first element of the output list consists of two 7x5 matrices, one for dams and one for sires. The columns are defined as follows, where by default Pedigree 1 is the older pedigree, and Pedigree 2 the newly reconstructed pedigree:

- Total: The total number of individuals with a parent assigned in either or both pedigrees. When Pedigree 1 is the true pedigree (in case of simulated data), this is the maximum number of matches.
- Match: The same parent is assigned in both pedigrees (non-missing). For dummy parents, it is considered a match if the inferred sibship containing the most offspring of a non-genotyped parent, consists for more than half of its offspring.
- Mismatch: Different parents assigned in the two pedigrees. When a sibship according to Pedigree 1 is split over two sibships in Pedigree 2, the smaller fraction is included in the count here.
- P1only: Parent present in Pedigree 1 but not assigned in 2; includes non-assignable parents such as non-genotyped parents with a single offspring and lacking genotyped grandparents.
- P2only: Parent assigned in Pedigree 2 but not present 1; if pedigree 1 is the true pedigree, these are false positives.

The rows indicate the various categories, subdivided into:

GG Genotyped individual, assigned a genotyped parent in either pedigree

GD Genotyped individual, assigned a dummy parent, or at least 1 genotyped sibling or a genotyped grandparent in Pedigree 1)

GT Genotyped individual, total

DG Dummy individual, assigned a genotyped parent (i.e., grandparent of the sibship in Pedigree 2)

DD Dummy individual, assigned a dummy parent (i.e., avuncular relationship between sibships in Pedigree 2)

DT Dummy total

TT Total total, includes all genotyped individuals, plus non-genotyped individuals in Pedigree 1, plus non-replaced dummy individuals (see below) in Pedigree 2

Potential dummy individuals according to Pedigree 1 are all non-genotyped individuals who have at least two genotyped offspring, or who have one genotyped offspring and a genotyped parent (i.e. who are 'inbetween' a grandparent–grandoffspring pair). Note that not all of these will be truly identifiable with Sequoia, for example when the true grandparent is a founder, and cannot be distinguished from full avuncular.

The second list element, 'MergedPed', is a side-by-side comparison of the two pedigrees, plus three columns (id.real, dam.real, sire.real) where dummy IDs in Pedigree 2 are replaced by the most likely non-genotyped individual from Pedigree 1.

This merged pedigree is collapsed in the third element, 'ConsensusPed', here with Sequoia assignments (assumed Pedigree 2) taking priority over the 'old' pedigree (Pedigree 1), and dummy parents being replaced where known (suffix '.r'). The columns 'dam.cat' and 'sire.cat' indicate with a 2-letter code whether the focal individual and the assigned parent were genotyped (G), a dummy individual in Pedigree 2 (D), a dummy individual replaced by a best-match non-genotyped individual from Pedigree 1 (R) or ungenotyped (U).

**Example** In the example included in the helpfile (`?sequoia`), pedigree reconstruction using the example genotype data included in the package (Simulated from the first 2 generations in Ped_HSg5.txt) results in two mismatches:

```
> data(SimGeno_example, LH_HSg5, Ped_HSg5)
> SeqOUTX <- sequoia(GenoM = SimGeno_example, LifeHistData = LH_HSg5)
> #
> # compare
> compare <- PedCompare(Ped1 = Ped_HSg5,
+                       Ped2 = SeqOUTX$Pedigree)
> compare$Counts
> # , , dam
> #    Total Match Mismatch P1only P2only
> # GG   130   127        2      1      0
> # GD    54    52        2      0      0
> # GT   182   179        2      1      0
> # DG     0     0        0      0      0
> # DD     0     0        0      0      0
> # DT     0     0        0      0      0
> # TT   960   179        2    779      0
> # ...
```

where the entries for DR, DD and DT are 0 because none of the dummy parents could have had a parent assigned - there are only 2 generations in this dataset.

We can investigate these mismatches further:

```
> with(compare$MergedPed,
+      compare$MergedPed[which(dam.1!=dam.2 & dam.1!=dam.r), ])
> #     id  dam.1 sire.1 dam.2 sire.2   id.r   dam.r sire.r
> # b01137 a00001 b00009 F0007 b00009 b01137 nomatch   <NA>
> # a01139 a00001 b00009 F0007 b00009 a01139 nomatch   <NA>
> #
> compare$MergedPed[which(compare$MergedPed$dam.2=="F0007"), ]
> # --> only a01139 and b01137 are in this half-sibship
> compare$MergedPed[which(compare$MergedPed$dam.1=="a00001"), ]
> # --> a01139 and b01137 are the only ones not assigned to a00001
> #
> SeqOUTX$MaybeParent
> #       ID1    ID2 Sex1 Sex2 AgeDif Relx LLR_Rx_U TopRel LLR_R1_R2 OH
> # (...)
> # 10 a01063 b00013    1    2      1   PO    10.63     FS      1.37  1
> # 3  a01139 a00001    1    1      1   PO     7.32    2nd      4.79  2 <--
> # 8  b01168 b00002    2    2      1   PO     4.05    2nd      1.23  2
> # 2  b01137 a00001    2    1      1   PO     8.90    2nd      0.91  1 <--
> # 6  b01165 b00002    2    2      1   PO     4.14    2nd      0.45  2
> # 1  a01142 a00018    1    1      1   PO     9.89    2nd      0.17  2
```

It seems that a00001 was more likely to be a parent than unrelated to both b01137 and a01139 (‘LLR_Rx_U’ > 0), but due to genotyping errors (the number of opposing homozygous loci is 1 with b01137 and 2 with a01139) and Mendelian sampling, they were even more likely to be 2nd degree relatives (‘TopRel’).

Additionally, we see on the second line of `SeqOUTX$MaybeParent` that during the parentage assignment step, a01063 and b00013 were more likely to be full siblings (FS) than parent-offspring (which they in fact are), and would even pass the threshold Tassign (= 1). However, a correct assignment is made in the final pedigree:

```
> SeqOUTX$Pedigree[SeqOUTX$Pedigree$id=="a01063", ]
> #        id   dam   sire LLRdam LLRsire LLRpair
> # 90 a01063 F0001 b00013   3.88    2.47    6.05
```

due to the use of age prior information (all siblings occur within the same cohort, while b00013 and a01063 are in different cohorts) and/or clustering of a01063 with its maternal siblings (assignment of dummy mother F0001).

**Colony** To compare Colony output with an existing pedigree, use:

```
> BestConfig <- read.table("Colony/file/file.BestConfig",
+                          header=T, sep="", comment.char="")
> PedCompare(PedFile1 = "ExistingPedigree.txt",
+            Ped2 = BestConfig)
```

## 3.2 Estimating confidence probabilities

The provided likelihood ratio between the assigned parent being the parent versus otherwise related to the focal individual, does not necessarily indicate how likely the assignment is correct. Due to random Mendelian sampling and genotyping errors, true full siblings may have a higher likelihood to be parent-offspring than full siblings, for example.

Pedigree-wide confidence probabilities can be estimated by

- simulating genotype data according to the reconstructed (or an existing) pedigree, imposing realistic levels of missingness and genotyping errors;
- reconstructing a pedigree from these simulated data;
- counting the number of mismatches between the 'true' pedigree, used as input for the simulated data, and this reconstructed pedigree.

When repeated at least 10–20 times, the mean error count divided by the total number of pedigree links provides an estimate of one minus the the confidence probability. Note that this can be rather time consuming, and will give an anti-conservative estimate as the simulations assume all SNPs are completely independent.

An example script:

```
> data(LH_HSg5, Ped_HSg5)
> ARER <- array(dim=c(10,7,2))
> for (x in 1:10) {
+   cat(x, "\t", format(Sys.time(), "%H:%M:%S"), "\n")
+   GM <- SimGeno(Ped = Ped_HSg5, nSnp = 100)
+   SimOUT <- sequoia(GenoM = GM,  LifeHistData = LH_HSg5, quiet=TRUE)
+   CountX <- PedCompare(Ped1 = Ped_HSg5, Ped2 = SimOUT$Pedigree)$Counts
+   ARER[x,,1] <- rowMeans(CountX[, "Match", ] / CountX[, "Total", ])
+   ARER[x,,2] <- rowMeans((CountX[, "Mismatch", ] +
+                           CountX[, "P2only", ]) / CountX[, "Total", ])
+ }
> dimnames(ARER) <- list(c(1:10), dimnames(CountX)[[1]], c("AR", "ER"))
> #
> # average assignment rate (AR) & error rate (ER) per category:
> round(apply(ARER, c(2:3), mean), 4)
```

To add confidence probability to the pedigree based on real data

```
> ConfProb <- 1 - round(colMeans(ARER[, , "ER"]),3)
> #
> PedC <- PedCompare(Ped1 = Ped_HSg5,
+                    Ped2 = SeqOUT$Pedigree)$ConsensusPed
```

```
> PedC$dam.prob <- ConfProb[as.character(PedC$dam.cat)]
> PedC$sire.prob <- ConfProb[as.character(PedC$sire.cat)]
> #
> # or, when assuming that replacement of dummies by IDs of
> # non-genotyped individuals is free from error,
> PedC$dam.prob <- with(PedC,
+                       ifelse(dam.cat=="GG",
+                              ConfProb["GG"],
+                              ifelse(dam.cat %in% c("GD", "GR"),
+                                 ConfProb["GD"],
+                                 ifelse(dam.cat %in% c("DG", "RG"),
+                                    ConfProb["DG"],
+                                    ifelse(dam.cat %in% c("DD", "DR",
+                                                          "RD", "RR"),
+                                       ConfProb["DD"],
+                                       NA)))))
```

## 3.3 Comparison pedigree-based and genomic relatedness

In absence of a previous pedigree, or when it is not obvious whether the previous or newly inferred pedigree is correct, one can compare the pairwise relatedness estimated from the pedigrees to a measure of genomic relatedness, estimated directly from the SNP data. The latter will vary around the pedigree-based relatedness even for a perfect pedigree due to Mendelian variance, but outliers suggest pedigree errors.

Genomic relatedness can be estimated for example using GCTA, `http://cnsgenomics.com/software/gcta/estimate_grm.html`, ideally using as all available markers (i.e., potentially many more than used for pedigree reconstruction). Pedigree relatedness can be calculated for example using the R package `pedantics`. As the number of pairs $p$ becomes very large even for moderate numbers of individuals $n$ ($p = n \times (n-1)/2$), additional packages are required to assist with merging (`data.table`) and plotting (`hexbinplot`). For example:

```
> Rel.snp <- read.table("GT.grm.gz")
> Rel.id <- read.table("GT.grm.id", stringsAsFactors=FALSE)
> Rel.snp[,1] <- as.character(factor(Rel.snp[,1], labels=Rel.id[,2]))
> Rel.snp[,2] <- as.character(factor(Rel.snp[,2], labels=Rel.id[,2]))
> names(Rel.snp) <- c("IID2", "IID1", "SNPS", "R.SNP")
> Rel.snp <- Rel.snp[Rel.snp$IID1 != Rel.snp$IID2,]
> #
> library(pedantics)
> PedStats <- pedigreeStats(SeqOUT$Pedigree[,1:3], graphicalReport=FALSE,
+                           includeA=TRUE)
> Rel.ped <- as.data.frame.table(PedStats$Amatrix)
> names(Rel.ped) <- c("IID1", "IID2", "R.seq")
> #
> library(data.table)
> Rel.snp <- data.table(Rel.snp, key=c("IID1", "IID2"))
```

```
> Rel.ped <- data.table(Rel.ped, key=c("IID1", "IID2"))
> Rel.gt <- merge(Rel.snp[,c(1,2,4)], Rel.ped, all.x=TRUE)
> Rel.gt <- as.data.frame(Rel.gt)
> rm(PedStats, Rel.snp, Rel.ped)
> #
> round(cor(Rel.gt[, 3:4], use="pairwise.complete"),4)
> #
> library(hexbin)
> ColF <- function(n) rev(rainbow(n, start=0, end=4/6,
+                                  s=seq(.9,.6,length.out=n),v=.8))
> hexbinplot(Rel.gt$R.SNP~Rel.gt$R.ped, xbins=100, aspect=1, maxcnt=10^6.5,
+            trans=log10,inv=function(x) 10^x, colorcut=seq(0,1,length=14),
+            xlab="Pedigree relatedness", ylab="Genomic relatedness",
+            xlim=c(-.1,.9), ylim=c(-.1, .9), colramp=ColF, colorkey = TRUE)
```

# References


[1] J Huisman. Pedigree reconstruction using snp data: parentage assignment, sibship clustering, and beyond. *Molecular Ecology Resources*, under review.

[2] T C Marshall, J B K E Slate, L E B Kruuk, and J M Pemberton. Statistical confidence for likelihood-based paternity inference in natural populations. *Molecular ecology*, 7(5):639–655, 1998.

[3] S Purcell, B Neale, K Todd-Brown, L Thomas, M A R Ferreira, D Bender, J Maller, P Sklar, PIW De Bakker, MJ Daly, et al. PLINK: a tool set for whole-genome association and population-based linkage analyses. *The American Journal of Human Genetics*, 81(3):559–575, 2007.

[4] E A Thompson and T R Meagher. Parental and sib likelihoods in genealogy reconstruction. *Biometrics*, pages 585–600, 1987.
